# Computational Investigation of Substituent Effects on the Alcohol + Carbonyl Channel of Peroxy Radical Self- and Cross-Reactions

**DOI:** 10.1021/acs.jpca.2c08927

**Published:** 2023-02-08

**Authors:** Galib Hasan, Vili-Taneli Salo, Thomas Golin Almeida, Rashid R. Valiev, Theo Kurtén

**Affiliations:** † Department of Chemistry, University of Helsinki, P.O. Box 55, 00014 Helsinki, Finland; ‡ Institute for Atmospheric and Earth System Research, Faculty of Science, University of Helsinki, 00014 Helsinki, Finland

## Abstract

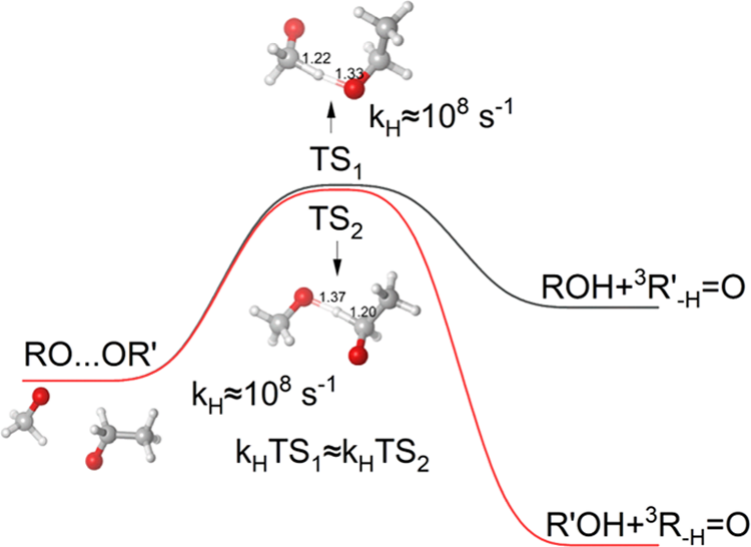

Organic peroxy radicals (RO_2_) are key intermediates in atmospheric chemistry and can undergo a large variety of both uni- and bimolecular reactions. One of the least understood reaction classes of RO_2_ are their self- and cross-reactions: RO_2_ + R′O_2_. In our previous work, we have investigated how RO_2_ + R′O_2_ reactions can lead to the formation of ROOR′ accretion products through intersystem crossings and subsequent recombination of a triplet intermediate complex ^3^(RO···OR′). Accretion products can potentially have very low saturation vapor pressures, and may therefore participate in the formation of aerosol particles. In this work, we investigate the competing H-shift channel, which leads to the formation of more volatile carbonyl and alcohol products. This is one of the main, and sometimes the dominant, RO_2_ + R′O_2_ reaction channels for small RO_2_. We investigate how substituents (R and R′ groups) affect the H-shift barriers and rates for a set of ^3^(RO···OR′) complexes. The variation in barrier heights and rates is found to be surprisingly small, and most computed H-shift rates are fast: around 10^8^–10^9^ s^–1^. We find that the barrier height is affected by three competing factors: (1) the weakening of the breaking C–H bond due to interactions with adjacent functional groups; (2) the overall binding energy of the ^3^(RO···OR′), which tends to increase the barrier height; and (3) the thermodynamic stability of the reaction products. We also calculated intersystem crossing rate coefficients (ISC) for the same systems and found that most of them were of the same order of magnitude as the H-shift rates. This suggests that both studied channels are competitive for small and medium-sized RO_2_. However, for complex enough R or R′ groups, the binding energy effect may render the H-shift channel uncompetitive with intersystem crossings (and thus ROOR′ formation), as the rate of the latter, while variable, seems to be largely independent of system size. This may help explain the experimental observation that accretion product formation becomes highly effective for large and multifunctional RO_2_.

## Introduction/Background

Organic peroxy radicals (RO_2_) are critical intermediates in the troposphere, and their reactions directly affect the formation of secondary organic aerosol (SOA). Aerosols, especially fine and ultrafine particles, have significant effects on air quality and health. Aerosols also directly affect the Earth’s energy budget by scattering and absorbing solar radiation, on average leading to cooling and warming effects, respectively.^1^ They also have an indirect effect by acting as nuclei for cloud and fog formation.^2^ The overall effect of aerosols on climate is believed to be cooling.

RO_2_ are produced by the oxidation of hydrocarbons, which are in turn emitted into the environment from both anthropogenic and biogenic activities.^3^ The oxidation process is initiated by a small set of oxidants (mainly OH, O_3_, and NO_3_), followed by O_2_ addition to form the peroxy radical intermediates (RO_2_).^4,5^

**Scheme 1:**
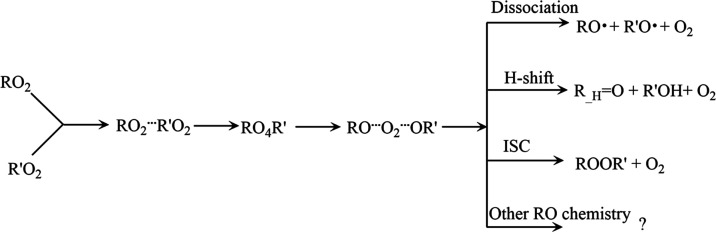
Schematic of the Mechanism for the Cross-Reactions Between Two Peroxy Radicals (RO_2_), and the Possible Product Channels

Recent computational^6^ and experimental studies^7^ have demonstrated that RO_2_ containing suitable (usually oxygen-containing) functional groups can undergo a series of sequential unimolecular hydrogen shift (H-shift) isomerization and oxygen addition reactions. This “autoxidation” can ultimately lead to products containing most of the carbon atoms of the parent hydrocarbon, and as many as 10 oxygen atoms.^8^ While bimolecular reactions of RO_2_ are in most atmospheric conditions dominated by reactions with NO_x_ and HO_2_, RO_2_ + R′O_2_ self- and cross-reactions are important side channels as they can, especially in the case of autoxidation-generated polyfunctional RO_2_, lead to aerosol-forming low-volatility accretion products. The RO_2_ + R′O_2_ reaction has three well-established channels (for the full mechanism, see Scheme 1)
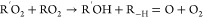
1
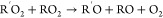
2
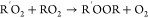
3

The “alcohol + carbonyl” route (reaction 1) is known to be one of the main, and sometimes the dominant, RO_2_ + R′O_2_ reaction channels for small RO_2_. However, few details about this reaction have been experimentally resolved, and the factors determining the branching ratios (e.g., this channel compared to the RO + R′O alkoxy channel, or the ROOR′ accretion product channel for larger RO_2_) are poorly understood. More complex RO may also have additional reaction channels available.

A recent theoretical study by Lee et al.^9^ comprehensively explains the fundamental mechanism of RO_2_ self-reactions. According to their studies, all RO_2_ + R′O_2_ reaction channels proceed through the same singlet RO_4_R′ tetroxide structure, which undergoes two O–O bond cleavages to form a singlet (RO···O_2_···R′O) cluster, where the O_2_ is in its triplet ground state, and the two ROs have the same spin. ROOR′ formation (reaction 3) is thus prevented not by an energy barrier, but by the need for a spin-flip (intersystem crossing, an effect originating from relativistic quantum mechanics). This may render the channel uncompetitive for most small RO_2_, as the relatively weak binding of the corresponding small ^3^(RO···OR′) clusters leads to their rapid dissociation.

Our previous modeling,^10^ along with some experimental evidence (e.g., the evidence for triplet carbonyl formation cited in Lee et al.^9^ and Ghigo et al.^6^) indicates that the mechanism for reaction 1 is an intermolecular H-shift from one alkoxy radical to another inside a triplet (RO···OR′) complex. While much literature data exist on unimolecular alkoxy H-shifts,^11^ there are, to our knowledge, no systematic studies on intermolecular alkoxy H-shifts, especially not on the triplet surface. We have a few data points from our previous paper,^9^ but nothing systematically exploring substituent effects.

Thus, we carry out a systematic study, varying substituents on both the “donor” and “acceptor” RO, and studying the H-shift barriers and rates. Here, the “donor” refers to the RO from which the H atom is abstracted, while “acceptor” refers to the RO doing the abstracting. We compute the energetics for reactions 1 and 2 for a set of small ^3^(RO···OR′) systems with substituents relevant to atmospheric chemistry, as well as to the experimental work of Berndt et al.^7^ The studied RO are methylalkoxy (MeO), ethylalkoxy (EtO), isopropoxy (iPrO), acetonylalkoxy (AceO), hydroxyisopropoxy (O*i*PrOH), and hydroxybutoxy (OBuOH). The latter two systems have the OH group on a terminal carbon atom, and the alkoxy group on an adjacent carbon, corresponding to the dominant products of OH-initiated alkene oxidation.

We carry out systematic conformational sampling on triplet potential energy surfaces for all of the possible combinations of ^3^(RO···OR′) systems with R = MeO, EtO, iPrO or AceO, and R ≠ R′, searching for both representative minimum-energy structures and for H-shift transition states leading to the formation of an alcohol and a ketone/aldehyde. Finally, for comparison, we compute intersystem crossing rate coefficients using a state-of-the-art multireference computational method. (For data on systems with R = R′, we refer to our previous study).^10^

## Theory and Methods

The details of our conformational sampling workflow have been presented in our previous work.^9^ Briefly, the alkoxy reactant molecules (“monomers”) were created in Spartan version 18,^12^ and the systematic conformer search algorithm was used to construct a complete set of monomer conformers. In this search, every torsional angle is considered, and every nonterminal bond is rotated (typically over 120°) using the Merck molecular force field (MMFF).

We also further modified the Jammy Key for Configurational Sampling (JKCS) approach^11^ and used it in this work to explore the complex potential energy surface of the triplet ^3^(RO···OR′) clusters. The distinct alkoxy monomer (reactant) conformers were taken from Spartan and optimized at the ωB97X-D/aug-cc-pVTZ level of theory using Gaussian16 RevB.01.^13^ This provided the monomer geometries for the subsequent cluster sampling. In our configurational sampling approach, we first use an artificial bee colony (ABC) algorithm to explore thousands of cluster conformers using rigid monomer structures in a molecular dynamic simulation.^14,15^ In addition to monomer structures, this algorithm requires Lennard-Jones parameters and partial charges for all atoms. Partial charges were obtained using natural bonding orbital (NBO) calculation at the ωB97X-D/aug-cc-pVTZ level, while Lennard-Jones parameters were collected from a force field database.^14,15^

In the ABC step, we initially generate 3000 cluster conformers for each pair of monomer conformers. Then, a semiempirical tight bonding optimization is performed using the *GFN-xTB* method (version GFN2).^16^ During our XTB optimization, various unwanted reactions (bond breaking and forming) sometimes occurred. Some of these reactions are likely artifacts of the XTB method, while others may correspond to actually available reaction channels (such as the H-shifts studied later). However, as the purpose of this initial sampling was to construct (unreacted) reactant geometries, these unwanted conformers were eliminated using the python-based GOODVIBES program.^17^ For each cluster, we used certain bond lengths and angles as threshold criteria to collect only intact (unreacted) conformers from the XTB run. We also eliminated all duplicate structures by considering dipole moments and the radius of gyration parameters, similarly to what was done in our previous work.^10^ We then took only the distinct and intact conformers within 15 kcal/mol in XTB energy and optimized them at the UωB97X-D/6-31+G(d) level of theory, followed by frequency analysis. We then performed another round of systematic filtering. All redundant structures were eliminated, and we considered further only the conformers within 5 kcal/mol of the lowest-energy conformer in the higher-level density functional theory (DFT) optimization. For the remaining conformers, we used U*ω*B97X-D/aug-cc-pVTZ level of theory for both optimization and vibrational frequency analysis using Gaussian16 RevB.01.^13,18−20^ We then took the single lowest-energy conformer, and performed a ROHF-ROCCSD(T)-F12a/cc-pVDZ-F12 energy calculation using Molpro 2021.^21−23^ (These CCSD(T)-F12 energy calculations—abbreviated from here on as F12—were also performed for the lowest-energy alkoxy monomers, as well as for the product molecules described below.)

Initial guesses for the H-shift transition state (TS) were created from the global minimum conformer of each ^3^(RO···OR′), and used as an input for the TS conformer sampling as described in our previous work.^9^ In brief, several C, H, and O bond lengths and angles (depending on the system) were constrained before the conformer search initiation since no conformer searching algorithm is available for transition state geometries. We then performed constrained conformer searching (rotating over all unconstrained torsions) with the MMFF method, followed by constrained optimizations, and subsequent full (unconstrained) TS optimizations at the UB3LYP/6-31+G(d) level of theory. We removed all duplicate structures (as described above), and selected conformers within 5 kcal/mol for TS optimizations at the UωB97X-D/aug-cc-pVTZ level. We also performed an Intrinsic Reaction Coordinate (IRC) calculation in both forward and reverse directions from the lowest-energy TS conformer to verify that the TS connects to the correct reactants and products, and to search for possible H-bonded product complexes. The IRC calculation was performed at the B3LYP level, and the IRC endpoints were finally optimized at the UωB97X-D/aug-cc-pVTZ level of theory. The highest spin contamination found in any of the transition states was 0.0165 before annihilation and 0.0001 after annihilation.

We also performed the conformer sampling of the separated product molecules with the same approach described above for the monomers.^9^ Since the H-shift reaction from ^3^(RO···OR′) can in principle give either ^3^R_–H_ = O + ^1^R′OH or ^1^R_–H_ = O + ^3^R′OH products, we performed the DFT calculations on both products at both singlet and triplet surfaces. While carbonyl compounds are well known to have low-lying triplet states, some of the “alcohol” products also have carbonyl functional groups. Thus, it is not always immediately obvious that the ^3^R_–H_ = O + ^1^R′OH combination will be much lower in energy. Calculations on triplet states of 1,2-propanediol and methanol did not converge and were thus omitted from the comparison.

All free energies and other thermodynamic parameters are calculated at 298.15 K and 1 atm reference pressure. The final cluster binding (free) energies, as well as the H-shift rates, are computed using the single lowest-energy conformers found in the sampling (for the reactants, transition states, and products). As discussed by Elm et al.,^24^ neglecting higher-energy conformers introduces a modest error source—likely on the order of 1 kcal/mol—to the calculation of cluster formation or dissociation free energies. As our reactant clusters very likely have more conformers than either the H-shift transition states or the (dissociation or H-shift) reaction products, our reaction free energies may thus be slightly biased in favor of the products, while our H-shift rates may be slightly too high. However, we note that for relatively weakly bonded clusters without strong specific interactions such as H bonds (e.g., the triplet complexes of two alkyl-RO studied here), it is quite difficult to determine when two almost-identical cluster structures produced in a quantum chemical optimization are genuinely different local minima that should be included, and when they are duplicates that should be discarded. If local minima were to be included in the calculations, two different (but still both entirely reasonable) settings for the duplicate screening stage could thus lead to potentially quite different results. For this reason, we have chosen to use only the single global minimum conformers in our final calculations, despite the modest error that this incurs.

During the coupled-cluster calculation, we had issues with Hartree–Fock (HF) convergence for some of the transition states. We anticipate that this problem is related to the ROHF solver converging to an incorrect (artificial) solution rather than the true minimum energy. For some cases, the difference between the DFT and F12 barrier heights (obtained using the standard HF solver in Molpro) was also significantly high, for example, 8–12 kcal/mol.

We tested several approaches to get rid of this problem, including random orbital rotations, and two different approaches based on the multiconfigurational self-consistent field (MCSCF) solver. First, we performed orbital rotations at the ROHF/cc-pVDZ-F12 level as suggested by Vaucher and Reiher.^25^ As recommended, we considered 100 completely separate orbital rotations by rotating 10 randomly picked pairs from the 15 highest occupied and 15 lowest unoccupied orbitals, performed ROHF calculations using the cc-pVDZ-F12 basis, and picked the lowest-energy solution. (The difference between the default HF energy and that obtained using rotated orbitals was up to 5–9 kcal/mol in our TS calculations, which demonstrates that the default HF solver certainly had converged to an incorrect solution.) The actual coupled-cluster calculation was then run using the lowest-energy HF solution found. At this point, some of the systems converged, while some still had convergence issues at the coupled-cluster stage. We next tested an approach where a complete active space self-consistent field CASSCF(2,2) calculation was run before the HF stage, allowing for “dynamic” selection of the rotations. Unfortunately, this often led to HF energies higher than those found using the random orbital rotation approach. We finally attempted an MCSCF approach using the MINAO minimal basis but a larger active space, obtained by freezing all 1s and 2s orbitals of heavy (non-H) atoms, but including all occupied p-orbitals/electrons of the C and O atoms, 1s orbitals/electrons of H atoms, and one virtual (unoccupied) orbital to allow for rotations within the active space. This approach worked (in the sense of finding the lowest HF solution found by any other approach) for all except two cases: TS_2_ of EtO···OAce and TS_1_ of *i*PrO···OBuOH (see below).

### Rate Coefficient Calculation

The unimolecular rate coefficients for the H-shift reactions ^3^(RO···OR′) ⇒ R′_–H_ = O + ROH were calculated with canonical lowest-energy conformer transition state theory (TST), as given in eq 4.^26^
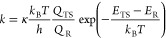
4where κ is the Eckart tunneling coefficient, *k*_B_ is the Boltzmann constant, *T* is the absolute temperature, *h* is Planck’s constant, and *Q*_TS_ and *Q*_R_ are the partition functions of the lowest-energy transition state and reactant, respectively. Finally, *E*_TS_ and *E*_R_ are the corresponding zero-point-energy corrected electronic energies of the transition state and the reactant. The activation barrier height (*E*_TS_ – *E*_R_) was calculated using DFT (UωB97X-D/aug-cc-pVTZ), both with and without ROHF-ROCCSD(T)-F12a/cc-pVDZ-F12 energy corrections.

Quantum mechanical tunneling can have a significant effect on H-shift rates, though due to the low barriers of the reactions studied here, the effect is smaller than for unimolecular RO or RO_2_ H-shifts. The Eckart tunneling approach^27^ has been suggested by Møller et al.^28^ as a cost-effective choice for H-shift reactions, as it shows good agreement with (much more expensive) multidimensional small curvature tunneling calculations.^29,30^ The forward and reverse barrier heights for the tunneling calculation were computed using the reactant and product conformers connected by IRC paths to the lowest-energy TS conformer.

### Intersystem Crossing Rate Calculations

Intersystem crossing (ISC) rate constants were calculated using a multireference approach since especially the final, open-shell singlet state of the ^1^(RO···OR′) cluster is almost impossible to describe accurately with single-reference methods. The calculation details were explained in our previous work.^10,31,32^ Briefly, the ISC rate was calculated using the following formula (eq 5)
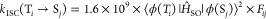
5where ⟨φ(T_*i*_)|*Ĥ*_SO_|φ(S_*j*_)⟩ is the spin–orbit coupling matrix elements (SOCME) between the initial triplet state (here, always T_1_) and the final singlet state in cm^–1^, and *F*_*ij*_ is Franck–Condon’s factor.^33^ Singlet and triplet energies were calculated using the XMC-QDPT2/6-311++G(d,p) level of theory in Firefly, version 8.2.0, 2016.^34^ The matrix element of the spin–orbit coupling interaction (SOCME) between T_1_–T_4_ and S_1_–S_4_ were calculated at the CASSCF level of theory but with the XMC-QDPT2(6,4)/6-311++G(d,p) energies as the zero-order energies within the perturbation theory.^33^ We used GAMESS-US for this calculation.^35^

## Results and Discussion

**Figure 1:**
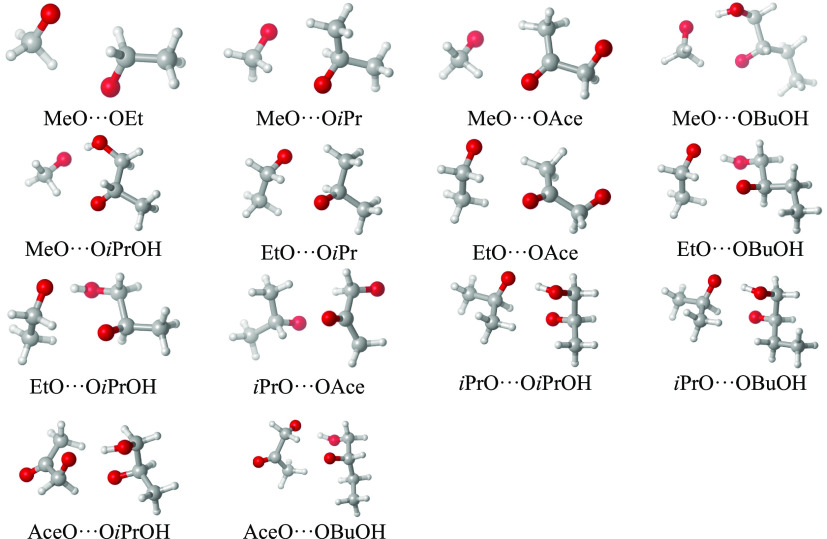
Optimized structures, at the ωB97X-D/aug-cc-pVTZ level, of the lowest-energy conformers of the ^3^(RO···OR′) clusters studied in this work. Color coding: gray = C, white = H, red = O.

The optimized minimum-energy structures of all ^3^(RO···OR′) clusters explored in this study are shown in Figure 1, and their binding energies (in kcal/mol, expressed in terms of dissociation reaction energies ^3^(RO···OR′) ⇒ RO + R′O) are given in Table 1. We note that the sign convention in Table 1 differs from that used, e.g., in studies of atmospheric molecular clustering, in which energies are typically given for the cluster formation reaction rather than for dissociation. The reason for our choice is that the ^3^(RO···OR′) ⇒ RO + R′O reaction is the one actually occurring in the atmosphere. The reverse reaction of RO + R′O colliding to form a cluster never happens due to the low concentration of alkoxy radicals. Positive values in Table 1 thus imply that the cluster is below the separated radicals in energy (or free energy), while negative values imply the opposite.

**Table 1:** Relative Electronic Energies (in kcal/mol) and Gibbs Free Energies (at 298 K and 1 atm Reference Pressure) for the Reaction Route ^3^(RO···OR′) ⇒ RO + R′O Computed at UωB97X-D/aug-cc-pVTZ (DFT) and ROHF-ROCCSD(T)-F12a/cc-pVDZ-F12 (F12) Levels of Theory

^3^(RO···OR′) cluster	Δ*E*_DFT_ (kcal/mol)	Δ*G*_DFT_ (kcal/mol)	Δ*E*_F12_ (kcal/mol)	Δ*G*_F12_ (=Δ*G*_DFT_ + Δ*E*_F12_ – Δ*E*_DFT_) (kcal/mol)	O···O distance in (Å)
MeO···OMe^a^	+3.32	–5.38	+3.15	–5.55	3.49
MeO···OEt	+4.57	–3.91	+7.07	–1.41	3.49
MeO···O*i*Pr	+3.70	–4.49	+3.16	–5.03	3.47
MeO···OAce	+3.34	–5.08	+3.63	–4.78	6.14
MeO···OBuOH	+6.99	–4.13	+6.05	–5.07	3.35
MeO···O*i*PrOH	+7.87	–3.21	+6.82	–4.27	3.35
EtO···O*i*Pr	+5.22	–4.17	+7.61	–1.79	3.59
EtO···OAce	+5.98	–2.83	+9.42	+0.60	4.21
EtO···OBuOH	+8.62	–2.48	+10.55	–0.54	3.27
EtO···O*i*PrOH	+9.49	–1.40	+11.29	+0.41	3.27
*i*PrO···OAce	+5.83	–4.11	+5.85	–4.09	4.16
*i*PrO···O*i*PrOH	+8.98	–2.12	+7.25	–3.85	3.31
*i*PrO···OBuOH	+8.33	–3.15	+6.24	–5.24	3.33
AceO···*i*PrOH	+9.59	–2.54	+7.52	–4.61	3.42
AceO···OBuOH	+9.90	–2.73	+7.66	–4.96	3.43

a MeO···OMe data from our previous study,^9^ provided here for reference and comparison.

The computed DFT binding energies (electronic energies, without zero-point energies) range from +3.34 to +9.90 kcal/mol, while the corresponding Gibbs free energies range from −5.08 to −1.40 kcal/mol. (The negative Gibbs free energies, especially when combined with the very low atmospheric concentration of free alkoxy radicals, imply that the hypothetical equilibria of the ^3^(RO···OR′) ⇒ RO + R′O reactions lie strongly on the side of the separated products. However, due to the high rates of the competing reactions for both the reactant and the products, this equilibrium will never have time to form in the atmosphere.) The DFT binding energies seem internally consistent, with, e.g., the presence of H-bonding functional groups typically leading to stronger binding, and chemically similar clusters having similar binding energies. The most stable cluster (at the DFT level) found in this study is AceO···OBuOH, with a binding energy of 9.90 kcal/mol. The reason for the strong binding is a hydrogen bond between the OH group of OBuOH and the alkoxy group of AceO. The C–H···O distance (between hydrogen and radical oxygen) is also quite small (1.8Å), indicating another attractive interaction. In general, all the clusters with H-bonding OH groups have binding energies above 6.9 kcal/mol (and typically contain H bonds to the alkoxy radical oxygen), while those without such groups have binding energies below 6 kcal/mol. Somewhat surprisingly, the weakest-bound complex in this study is MeO···OAce, with a binding energy value that is very similar to the one found for the MeO···OMe cluster in our previous study.^9^ The other complexes involving an AceO and an alkyl-substituted RO partner (EtO and **i**PrO) are among the strongest-bound clusters lacking OH groups. Together with the cluster structures shown in Figure 1, this suggests that interactions between the ketone group and the H atoms bonded to the alkoxyl carbon (α-oxyl C) are substantially weaker than those between the ketone and H atoms bonded to alkyl carbons (β-oxyl C). In general, adding alkyl groups tends (with some exceptions) to slightly increase binding energies, likely due to the increasing number of (individually weak) CH···HC and CH···O interactions.

The distance between the position of alkoxy radical centers ranged from 3.27 to 6.14 Å. The larger distances (and especially the 6.14 Å outlier) correspond to the AceO-containing clusters, as they—apart from the H-bonding cases—tend to interact with the other RO primarily via the ketone group. In the studied dataset, there is a weak inverse relationship between the radical distance and the binding energy, as the weakest-bonded AceO···alkyl-RO clusters have the longest distances. However, all other clusters have radical center distances of less than 3.6 Å.

In our previous work^9^ including mostly “homodimers” (i.e., ^3^(RO···OR′) clusters with identical R and R′) of a smaller set of more complex RO, we noticed considerable inconsistencies between DFT and F12 binding energies. Surprisingly, the F12 energies in this work are mostly consistent with the DFT energies and follow almost the same trends, though with a larger variation. The 4 kcal/mol difference between MeO···OEt and MeO···O*i*Pr, however, is reminiscent of the issues encountered in our previous paper (where minuscule structural differences lead to enormous differences in F12 energies). As in our previous study, we performed orbital rotations as suggested by Vaucher and Reiher^25^ also on the ^3^(RO···OR′) minima. However, in contrast to the transition states discussed earlier, this did not lead to lower HF energies. As the main topics of this study are the H-shift reactions, and as the numerous convergence problems in the F12 transition state calculations indicate severe problems with these approaches (despite the relatively low values of standard multireference diagnostics such as D1, T1, and %TAE(T)^36,37^ as shown in our previous paper), we focus our discussion and analysis on the DFT energetics and provide the F12 results for reference only. We note that, for example, Møller et al.^28^ have adopted an even stronger approach (for example omitting or discarding F12 energy corrections altogether) for a related class of reactions; “scrambling” H-shifts between peroxy/hydroperoxide and alkoxy/alcohol functional groups.

### Transition States and TST Rates for the H-Shift Reactions

**Figure 2:**
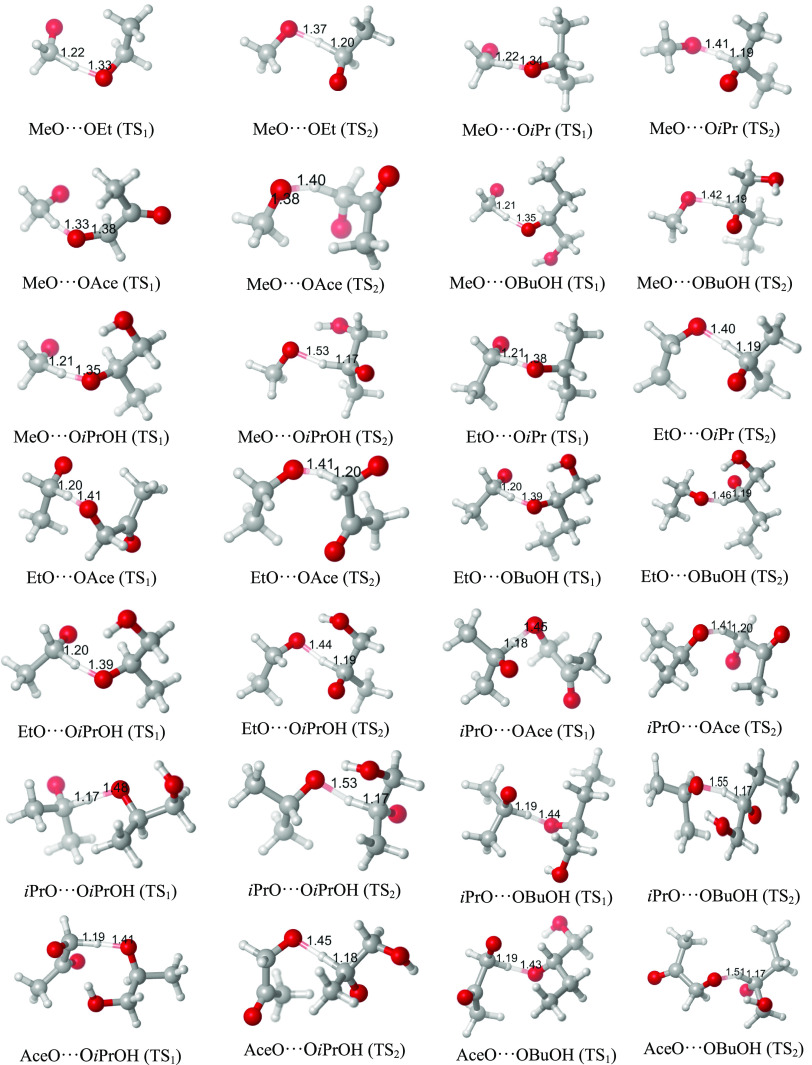
Transition state geometry for different ^3^(RO···OR′) systems, optimized at the Uωb97X-D/aug-cc-pVTZ level. Here, the distances between C–H and O–H bonds that are breaking and forming are given in angstrom (Å). Color coding: gray = C, white = H, red =O.

The lowest-energy transition state geometries are shown in Figure 2. As each cluster has two distinct alkoxy C atoms from which H atoms can be abstracted, two H-shifts (denoted TS_1_ and TS_2_) are shown for each cluster. For a ^3^(RO···OR′) cluster, TS_1_ denotes the H-shift reaction where RO acts as the donor and R′O as the acceptor of the shifting hydrogen atom, while TS_2_ denotes the reaction where RO is the acceptor and R′O the donor. The calculated barrier heights and H-shift reaction rates are given in Table 2 at both DFT and CCSD(T)-F12 levels. Tunneling coefficients corresponding to the DFT data are also given. For illustrations of H-shift transition states of the ^3^(MeO···OMe), ^3^(EtO···OEt), ^3^(*i*PrO···OiPr), and ^3^(AceO···OAce) homodimers, see our previous study.^9^

**Table 2:** Barrier Heights at Δ*E*_DFT_ and Δ*E*_CCSD(T)_ (Including Zero-Point Corrections), Tunneling Coefficient, and TST Rate Coefficients (at 298 K) at UωB97X-D/aug-cc-pVTZ (DFT) and ROHF-ROCCSD(T)-F12a/cc-pVDZ-F12 (F12) Levels of Theory

^3^(RO···OR′) cluster	TS	barrier height Δ*E*_DFT_ kcal/mol	barrier height Δ*E*_F12_ kcal/mol	tunneling factor (κ)	H-shift rate, using Δ*E*_DFT_, s^–1^	H-shift rate, using Δ*E*_F12_, s^–1^
MeO···OEt	TS_1_	5.21	7.73	4.48	2.15 × 10^8^	3.01 × 10^6^
	TS_2_	5.08	7.45	2.57	4.37 × 10^8^	8.03 × 10^6^
MeO···O*i*Pr	TS_1_	5.24	7.97	5.95	1.86 × 10^8^	1.88 × 10^6^
	TS_2_	3.69	5.79	1.47	1.43 ×10^9^	4.08 × 10^7^
MeO···OAce	TS_1_	4.89	6.97	4.84	2.48 × 10^8^	7.32 × 10^6^
	TS_2_	3.29	8.32	2.77	4.43 × 10^8^	9.10 × 10^4^
MeO···OBuOH	TS_1_	6.10	7.76	3.67	1.95 × 10^8^	1.16 × 10^7^
	TS_2_	5.67	7.74	1.46	3.37 × 10^8^	1.02 × 10^7^
MeO···O*i*PrOH	TS_1_	6.68	8.27	4.19	5.24 × 10^8^	3.59 × 10^7^
	TS_2_	5.69	7.99	1.19	7.10 × 10^7^	1.44 × 10^6^
EtO···O*i*Pr	TS_1_	4.28	6.77	3.43	6.85 × 10^8^	1.03 × 10^7^
	TS_2_	2.89	4.90	1.61	5.28 × 10^9^	1.77 × 10^8^
EtO···OAce	TS_1_	6.02	8.16	5.06	1.24 × 10^7^	3.33 × 10^5^
	TS_2_	5.98	12.30	2.16	7.30 × 10^7^	1.70 × 10^3^
EtO···OBuOH	TS_1_	9.64	10.50	2.47	3.41 × 10^6^	8.04 × 10^5^
	TS_2_	4.10	6.79	1.48	6.86 × 10^8^	7.29 × 10^6^
EtO···O*i*PrOH	TS_1_	6.74	8.04	2.12	2.51 × 10^8^	2.80 × 10^7^
	TS_2_	4.72	7.18	1.69	3.53 × 10^8^	5.52 × 10^6^
*i*PrO···OAce	TS_1_	4.87	6.43	1.35	1.22 × 10^8^	8.67 × 10^6^
	TS_2_	4.48	9.28	2.97	7.45 × 10^8^	2.29 × 10^5^
*i*PrO···OPrOH	TS_1_	8.33	9.09	1.16	1.43 × 10^6^	3.94 × 10^5^
	TS_2_	5.26	6.87	1.22	1.14 × 10^8^	7.62 × 10^6^
*i*PrO···OBuOH	TS_1_	4.65	14.21	1.46	5.99 × 10^8^	5.89 × 10^1^
	TS_2_	5.44	6.32	1.40	1.70 × 10^8^	1.11 × 10^7^
AceO···O*i*PrOH	TS_1_	5.21	5.94	2.98	3.56 × 10^8^	1.04 × 10^8^
	TS_2_	7.18	6.90	1.38	1.62 × 10^7^	2.62 × 10^7^
AceO···OBuOH	TS_1_	7.63	8.94	1.94	9.61 × 10^6^	1.05 × 10^6^
	TS_2_	5.70	6.32	1.16	1.93 × 10^8^	6.78 × 10^7^

The overall H-shift reaction energies, with four different combinations for each reactant corresponding to two reaction pathways, and two combinations of product multiplicities per pathway, are given in Section S3, with potential energy surfaces corresponding to the lower-energy product combinations shown in Section S4. For all except one case, the lowest-energy combination of products corresponded to ^3^R_–H_=O + ^1^R′OH, as expected given that carbonyl compounds tend to have relatively low-lying triplet states. The sole exception corresponded to AceO and OBuOH reacting to give 1-hydroxy-2-butanone and 1-hydroxyacetone, where the ^1^R_–H_=O + ^3^R′OH pair was 0.3 kcal/mol lower in energy than ^3^R_–H_=O + ^1^R′OH. This seemingly anomalous result is explained by the fact that in this case, both of the reaction products have carbonyl groups. (The question of whether the transition state for this system actually connects to ^1^R_–H_=O + ^3^R′OH rather than ^3^R_–H_=O + ^1^R′OH is beyond the scope of this study—this would require a rather complicated electron rearrangement unlikely to be well described by DFT methods.) In general, the reaction energies leading to ^3^R_–H_=O + ^1^R′OH can be divided into three groups. First, the reactions where the carbonyl product is formaldehyde (HCHO; i.e., where the H atom has been abstracted from a MeO alkoxy radical) are the least favorable, with reaction energies close to zero (varying from +3 to −1 kcal/mol). Second, reactions where the carbonyl product is methylglyoxal (i.e., where the H atom has been abstracted from an AceO alkoxy radical) are—presumably due to the low-lying triplet state of methylglyoxal—the most favorable, with reaction energies between −19 and −26 kcal/mol. (α-dicarbonyl compounds such as methylglyoxal are well known to have even lower triplet excitation energies than monocarbonyl compounds.) Third, all of the remaining reactions have energies between −3 and −8 kcal/mol. As entropic effects strongly favor the product side of the H-shift reaction, all of the H-shift reactions have negative Gibbs free energies.

All of the calculated H-shift rate coefficients (Table 2) were between 1 × 10^6^ and 5 × 10^9^ s^–1^ at the DFT level (with most values between 10^8^ and 10^9^ s^–1^) and between 6 and 2 × 10^8^ s^–1^ at the F12 level. The corresponding barrier heights ranged from 2.89 to 9.64 kcal/mol and from 4.90 to 14.21 kcal/mol, respectively. The DFT and F12 barrier heights were mostly consistent with each other, with the latter typically 2–3 kcal/mol higher than the former. In some cases, e.g., TS_2_ of EtO···OAce and TS_1_ of *i*PrO···OBuOH, the F12 barrier was much higher (by almost 6 and 9 kcal/mol, respectively). We note that these two systems correspond to the most problematic cases with respect to HF convergence (as discussed above), and caution that their F12 barriers are very likely highly unreliable. We further anticipate that the severe convergence issues we encountered are probably indicative of problems with the CCSD(T) calculations on all of these systems, and hence do not discuss these energetics or rates further.

The variation in (DFT) barrier heights and predicted rates is remarkably small compared to the corresponding variation in unimolecular alkoxy radical H-shift barriers and rates, as compiled for example in the seminal structure–activity relationship (SAR) by Vereecken et al.^38^ In this SAR, the barrier (and rate) of a RO H-shift is determined primarily by two factors: the “span” (i.e., the number of atoms between the shifting H atom and the accepting O atom in the transition state), and the substituents to the carbon atom from which the H atom is abstracted (i.e., the H-shift “donor”). In contrast, substituents around the “acceptor” RO group itself (to which the H atom is transferred) are in general (apart from the case of acyl alkoxy radicals) not accounted for by the SAR, as their effect is assumed to be minor.

**Figure 3:**
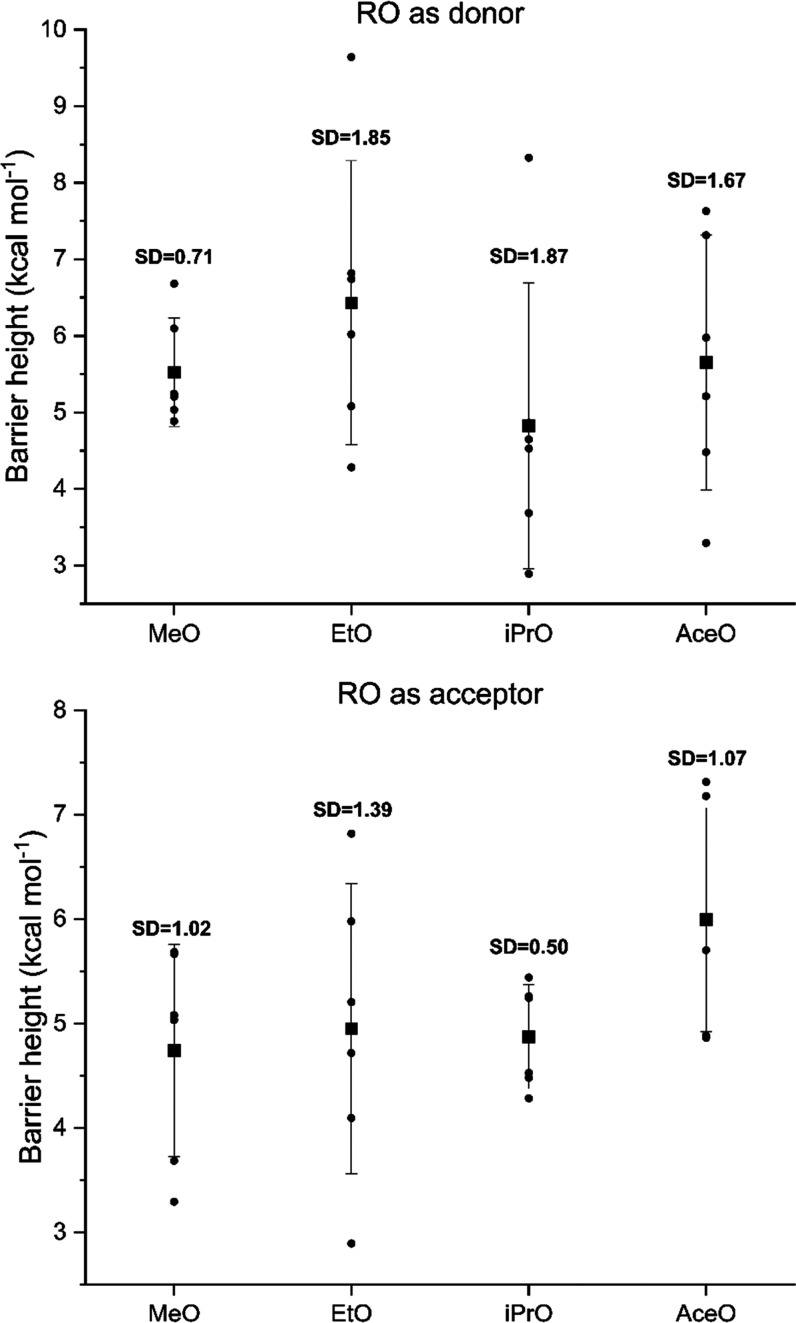
Top: H-shift barrier trends by grouping all of the donor RO. Bottom: H-shift barrier trends by grouping all of the acceptor RO. SD: standard deviation. All values correspond to UωB97X-D/aug-cc-pVTZ calculations.

To investigate whether a similar pattern holds also for our intermolecular H-shifts, we first grouped the transition states both by donor and acceptor (the donor being the RO from which the H atom is abstracted, and the acceptor being the RO abstracting the H atom), giving 6 data points for each type of donor and acceptor (when R=R′ homodimer data from our previous study is included). The spread in barrier heights for each donor and acceptor is shown in Figure 3. The results are unexpected: in three out of four cases the spread in barrier heights is larger for the donor than for the acceptor.

To facilitate a more direct comparison of the intermolecular and unimolecular cases, we used the Vereecken et al. SAR to compute reference values for each of our barrier heights. This was done as follows. First, since the carbon atoms of our donor and acceptor groups are not covalently bonded, the concept of “span” does not really apply to the intermolecular case. This lack of covalent ring strain is likely a major explanation for both the lower absolute values and the lower variability of the intermolecular H-shift barriers, compared to the intramolecular analogues. Thus, we used the SAR data for the least strained 1,5 span to compute the reference barrier and used the “exo” substituent corrections for the oxygen-containing functional groups (as the “endo” corrections correspond to substituents on the ring). Next, to match our three classes of C atom substitution to three classes available in the SAR, we compared our methyl donors (i.e., TSs where the H being abstracted is on MeO) to the “primary” cases in the SAR, our primary donors (where the H being abstracted is on EtO and AceO) to the “secondary” cases in the SAR, and our secondary donors (where the H being abstracted is on iPrO, HO-*i*PrO or HO-BuO) to the “tertiary” cases in the SAR. (Without this shift, two substitution classes in either dataset would need to be grouped together, as the SAR does not contain data on abstraction from CH_4_, while our dataset does not contain tertiary RO donors due to the lack of abstractable α-oxyl H atoms). Finally, the ketone and hydroxyl substituents on the AceO and HO-*i*PrO or HO-BuO donors were classified according to the SAR into exo-ß-oxo and exo-ß-hydroxy, respectively, and the reference barrier height was modified by the appropriate amount. Thus, all abstractions from MeO, EtO, and *i*PrO donors are given SAR reference barriers of 7.60, 5.98, and 4.85 kcal/mol (corresponding to the activation energy corrections for primary, secondary, and tertiary 1,5 H-shifts in Table 3 of the SAR, respectively). Abstractions from AceO were, like those from EtO, given a SAR reference barrier of 5.98 (as the correction factor corresponding to an exo-ß-oxo group, in Table 5 of the SAR, happens to be zero due to the way the SAR is set up). Finally, abstractions from HO-*i*PrO or HO-BuO donors are both given SAR reference barriers of 4.35 kcal/mol (obtained by adding the “tertiary” correction from Table 3 of the SAR and the “exo-ß-hydroxy” correction from Table 5 of the SAR).

**Figure 4:**
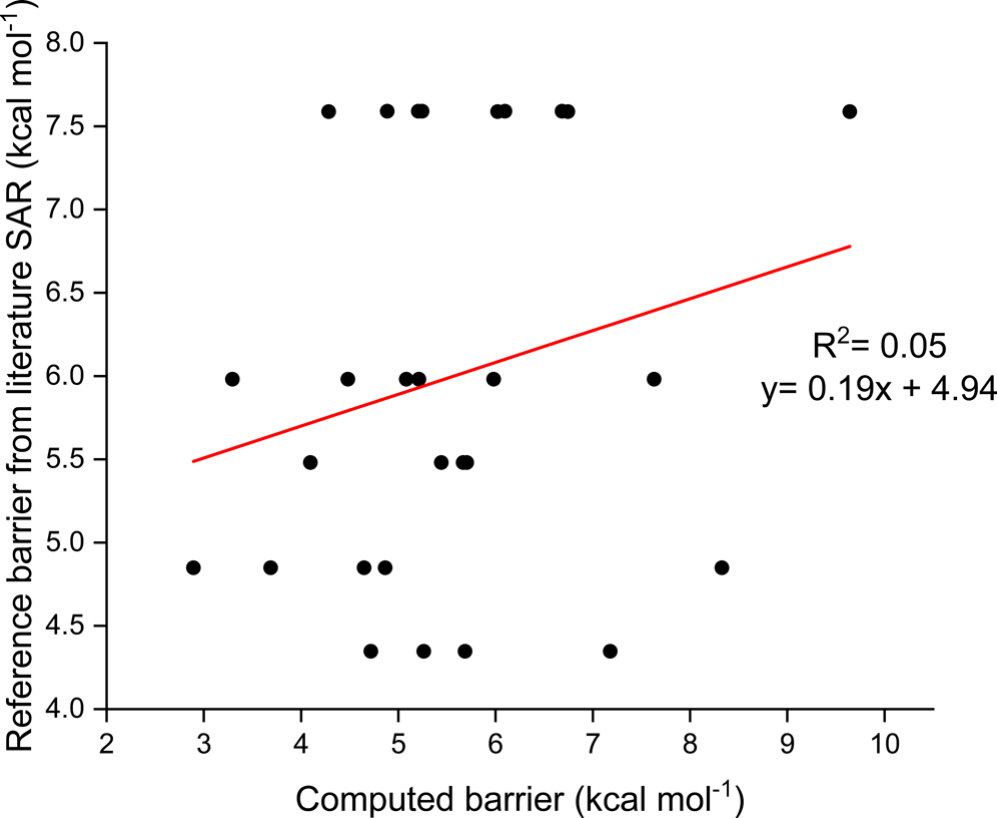
Reference barrier height (from a literature SAR on unimolecular RO H-shifts) vs the computed ^3^(RO···OR′) H-shift barrier height, at the UωB97X-D/aug-cc-pVTZ level.

The comparison is shown in Figure 4.

As expected already from the large spreads in barrier heights for the same H-atom donor (Figure 3), the correlation between the computed barrier heights and the corresponding unimolecular “reference” numbers is almost nonexistent. (The clustering of the SAR reference barriers into a few sets of horizontal lines in Figure 4 is a consequence of the SAR only treating substituents on or directly adjacent to the donor C atom, which leads to multiple H-shifts having the same reference barrier as described above.)

**Figure 5:**
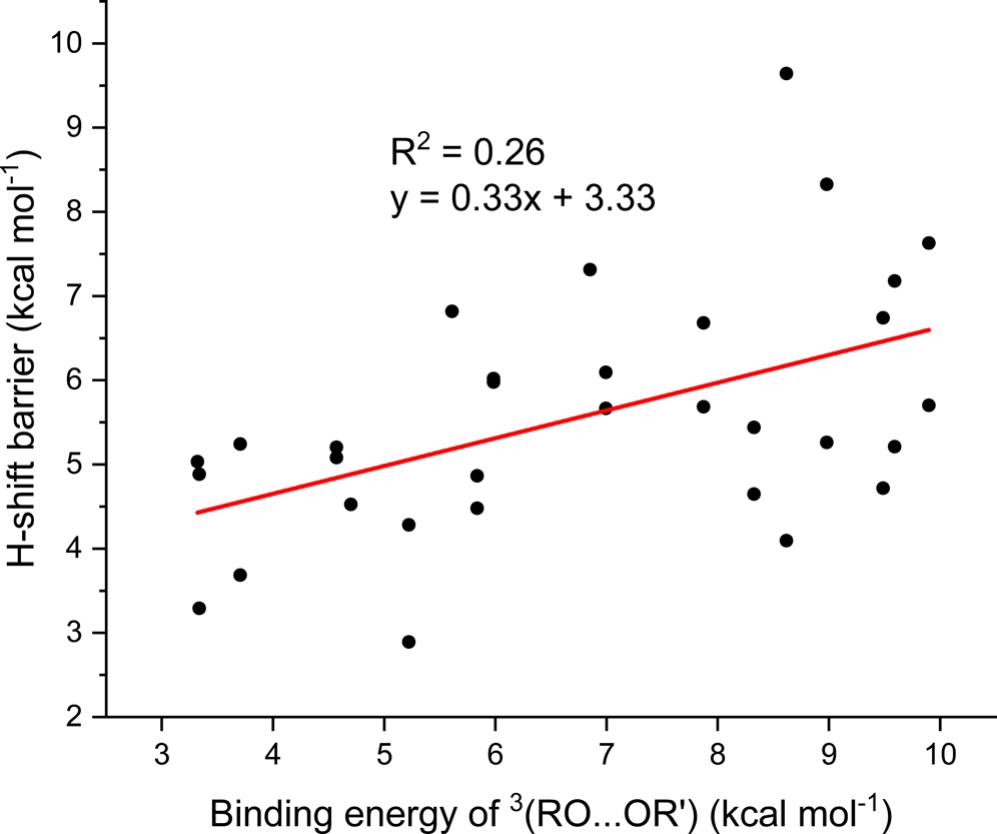
^3^(RO···OR′) H-shift barrier height vs the binding energy of the ^3^(RO···OR′) complex, at the UωB97X-D/aug-cc-pVTZ level.

Next, we investigated the dependence of the computed barrier heights (Table 2) on the ^3^(RO···OR′) binding energies (Table 1). This relationship is plotted in Figure 5. While the data are still scattered, there is a correlation between the two: an increase in binding energy by 1 kcal/mol will, on average, increase the H-shift barrier by 0.3 kcal/mol. The likely explanation for this is that forming the H-shift transition state requires breaking, or at least weakening, some of the bonding patterns in the minimum-energy ^3^(RO···OR′) complex, such as H bonds in the OH-containing systems. This energy penalty thus plays a role analogous to that of the ring strain in unimolecular H-shifts (typically expressed in terms of span-dependent barrier heights). The dependence of the H-shift barrier height on the ^3^(RO···OR′) binding energy also explains at least part of the initially puzzling dependence of H-shift rates on the H-acceptor RO: functional groups on the acceptor that increase the binding energy will tend to decrease the rate.

To partially remove the effects of the ^3^(RO···OR′) binding energy on the barrier heights, we recomputed all TS energies with respect to the hypothetical free RO + RO′ radical pair. We emphasize that these values are provided for reference and diagnostic purposes only: the collision of two separated alkoxy radicals will never happen in real atmospheric conditions. These barrier heights, and versions of Figures 3 and 4 computed using them, are provided in Section S2. While the scatter is still large, the correlation of the barriers with respect to free alkoxy radicals with the unimolecular SAR is improved, and the spread in barrier heights is now smaller for the donors than for the acceptors. However, the absolute spread of the barriers computed with MeO as the donor actually increases (compared to Figure 3), suggesting that more factors are still needed to explain the observed trends in barrier heights. Also, we note that when the effects of ^3^(RO···OR′) binding energy are removed, the barrier heights are all between about +3.5 and −3.5 kcal/mol. In other words, when computed with respect to the hypothetical RO + RO′ reference, all our H-shift reactions are quite close to barrierless, and the observed variation in barrier heights is only a few times larger than the likely error margin of the quantum chemical method, which is probably at least 2 kcal/mol given the difficulties in treating transition states of triplet clusters of two radicals. Our discussion on H-shift trends must thus be accompanied by a substantial caveat: much of the observed variation may be explained simply by random errors of the quantum chemical methods.

One possible reason for the scatter remaining in the data even after the cluster binding energies are subtracted is illustrated by the EtO···OBuOH system, which has the largest difference between the barriers of the two possible H-shift pathways of the studied systems (with TS_1_ almost 5.5 kcal/mol above TS_2_, despite the same cluster binding energy). Comparing the minimum-energy geometry of ^3^(EtO···OBuOH) from Figure 1 to the TS geometries in Figure 2, we can see that forming TS_1_ requires considerably more structural rearrangement than forming TS_2_, as the EtO oxygen atom in TS_1_ must be completely reoriented. In addition, we expect (as per the unimolecular SAR) that abstraction of a H atom on a more highly substituted carbon (such as that on OBuOH compared to EtO) will be at least somewhat easier even in the intermolecular case, further increasing the TS_1_–TS_2_ energy difference. The potential energy surfaces of the reactions (see Section S4) suggest yet another factor at play: the transition state corresponding to the pathway with lower product energies is, with one exception, always lower than the transition state corresponding to the pathway with higher product energies. (The exception is the *i*PrO···O*i*PrOH system, where the product energies differ by less than 0.7 kcal/mol, and where the higher-energy transition state requires much more rearrangement compared to the minimum-energy structure, similarly to the EtO···OBuOH case discussed above). The studied systems thus seem to follow a Bell–Evans–Polanyi-type relationship, where at least part of the barrier height is determined by the reaction energy. As the reaction energy strongly depends on the singlet-triplet gap of the product carbonyls, it is unsurprising that models and rules-of-thumb developed for ground-state unimolecular RO H-shifts fail to explain all of our data.

Our overall tentative hypothesis for the mechanisms governing the ^3^(RO···OR′) H-shift barriers is thus as follows. First, adding substituents to the donor RO tends to weaken the C–H bond strength (and decrease the H-shift barrier) just as in the case of unimolecular H-shifts. Second, substituents on both the donor and acceptor RO tend to increase the ^3^(RO···OR′) binding energy, which in turn increases the H-shift barrier, albeit with substantial variation depending on the degree of structural rearrangement required for the reaction. Third, channels leading to lower-energy products tend to have lower barriers, and this effect typically governs the relative ordering of the two possible transition states for an asymmetric (RO···OR′) system. (The third and the first effects are partially overlapping, as weaker reactant C–H bonds often go hand-in-hand with more stable H-abstraction products.) As especially the first two effects often counteract each other (with the same functional group simultaneously weakening the C–H bond being broken and increasing the cluster binding energy), making any quantitative predictions from our data is exceedingly difficult. However, we can make two qualitative predictions that should in principle be experimentally verifiable (or falsifiable). First, for many asymmetric systems (where R ≠ R′), the predicted rates for the two H-shift channels are quite close to each other. Excluding systems containing MeO (as thermodynamic constraints may prevent HCHO formation in these), for example, the EtO···O*i*PrOH and *i*PrO···OBuOH systems (formed in EtO_2_ + *i*PrOH-O_2_ and *i*PrO_2_ + BuOH-O_2_ peroxy radical cross-reactions, respectively) should form measurable yields of both possible carbonyl/alcohol pairs. Especially in the former, this would validate the hypothesis that substituent effects on donor C–H bond strengths are not the sole feature determining these H-shift rates. Second, as the R and R′ groups grow more complex and functionalized, the ^3^(RO···OR′) binding energy will tend to increase, in principle without an upper limit as more and more bonding interactions form. In contrast, the effect of functionalization beyond the carbon atoms adjacent to the RO group is unlikely (as per the unimolecular SAR) to substantially decrease the donor C–H bond strength further. Thus, we predict that for large or complex enough R or R′ groups, the binding energy effect will render the H-shift channel uncompetitive with intersystem crossings, as the rate of the latter, while highly variable, seems to be largely independent of system size (as shown below, and in our previous studies^9^). This mechanism likely helps explain the experimental indications that the formation of accretion products occurs at close to the kinetic limit for, e.g., self- and cross-reactions of polyfunctional monoterpene-derived RO_2_.^39^

### ISC Rate Calculation

While the focus of this study is on the mechanisms determining the barrier height and rate of the alcohol + carbonyl channel, we have for comparison, and to extend the dataset for our previous studies,^10,31,32^ also computed data for the intersystem crossing (ISC) reaction potentially leading to ROOR′ accretion product formation on the singlet surface. (As discussed above, recombination of the triplet radical pair to ROOR′ is forbidden by the Pauli principle.) The ISC rates for all the studied ^3^(RO···OR′) clusters are given in Section S5, with individual state-specific data given in Section S6.

The ISC rates for the set of systems studied here are roughly similar to those obtained earlier for RO_2_/RO derived from OH and NO_3_ -initiated *α*-pinene oxidation, and vary between about 10^8^ and 8 × 10^9^ s^–1^. They are thus also surprisingly similar to the H-shift rates in Table 2, suggesting (especially considering the likely order-of-magnitude error margins on both sets of rates) that for these small model systems, H-shifts and ISCs should typically both be competitive processes. The zero yields reported for ROOR′ formation in self- and cross-reactions of the smallest alkyl-RO_2_^5^ can likely be explained by a combination of energy nonaccommodation leading to the scission of ROOR′ with too few vibrational modes, and possible competing reactions (such as H-shifts) occurring also on the singlet surface. We caution that the ISC rate is only an upper limit: ROOR′ formation cannot happen without an ISC, but an ISC does not necessarily guarantee ROOR′ formation.

The present results further confirm that the extreme ISC rate values (4 × 10^3^ and 5 × 10^12^ s^–1^) found in our first study (using computational method essentially identical to those employed here) are indeed outliers and that the representative timescale for ^3^(RO···OR′) ISCs is measured in nanoseconds (rather than, e.g., pico- or milliseconds). As in our previous studies, the ISC rates are primarily determined by the spin–orbit coupling matrix elements (SOCME) between the T_1_ and S_1_ or S_2_ states. The energy gaps between T_1_ and S_1_ for the ^3^(RO···OR′) minimum-energy geometries are invariably low, and thus high SOCME values between these always lead to high ISC rates. In cases where the SOCME between T_1_ and S_1_ is low but that between T_1_ and S_2_ is high, ISCs to the excited singlet state S_2_ can also have high rates despite moderate (on the order of 1500–2500 cm^–1^) energy gaps.

## Conclusions

Self- and cross-reactions of peroxy radical compounds (RO_2_ + R′O_2_) play a significant role in atmospheric chemistry. In our previous studies, we have focused especially on the reaction channel potentially leading to ROOR′ accretion products. Here, we systematically study the effects of substituents on a competing channel leading to molecular (carbonyl and alcohol) products. Based on previous computational evidence, the key step for this channel is an intermolecular H-shift inside a triplet intermediate complex of two alkoxy radicals, ^3^(RO···OR′). We thus performed extensive conformational sampling to obtain both minimum-energy geometries and H-shift transition states for a total of 14 ^3^(RO···OR′) systems, each with two possible H-shift reaction pathways. We then computed rates for these pathways using transition state theory. The obtained rate coefficients displayed surprisingly small variation, with most H-shift rates being higher than 10^8^ s^–1^ but lower than 10^9^ s^–1^. The effect of functional groups on the H-shift rates turned out to be very difficult to explain, and our best hypothesis for rationalizing the (relatively modest) variation in computed barrier heights involves a competition between three different effects: the weakening of C–H bonds due to adjacent functional groups, the binding energy of ^3^(RO···OR′) tending to raise the barrier height due to the energy penalty required for cluster rearrangement to the TS geometry, and the product energy affecting the barrier in a Bell–Evans–Polanyi relationship. Intersystem crossing (ISC) rates computed for the same ^3^(RO···OR′) systems were of the same order of magnitude as the H-shift rates, suggesting that the two studied channels should be competitive for small and medium-sized systems. For larger and more complex systems, we predict that the increase in ^3^(RO···OR′) binding energy will tend to raise H-shift barriers, and ultimately render the alcohol + carbonyl pathway uncompetitive.

## Supporting Information Available

Additional H-shift barrier trends, overall reaction energies, potential energy surfaces, and detailed ISC rate data (PDF)
Output files of all quantum chemical calculations used to generate the data in this manuscript (ZIP)
