# Supplementary material for: Computational Investigation of Substituent Effects on the Alcohol + Carbonyl Channel of Peroxy Radical Self- and Cross-Reactions
Source: J Phys Chem A. 2023 Feb 8;127(7):1686–96. doi: 10.1021/acs.jpca.2c08927 (PMC9969516; doi:10.1021/acs.jpca.2c08927)
Supplement: Supplementary file 1 — jp2c08927_si_001.pdf [file jp2c08927_si_001.pdf]

# Supplementary Information for "Computational Investigation of Substituent Effects on the Alcohol + Carbonyl Channel of Peroxy Radical Self- and Cross Reactions

Galib Hasan[a,b*], Vili-Taneli Salo[a,b], Thomas Golin Almeida[a,b], Rashid R. Valiev[a,b], and Theo Kurtén[a,b*]

[a]Department of Chemistry, University of Helsinki, POB 55, FIN-00014 Helsinki, Finland

[b]Institute for Atmospheric and Earth System Research, Faculty of Science, University of Helsinki, Helsinki 00014, Finland

## Contents

**S1: H-shift barrier trends by grouping all donor and acceptor RO**

**S2: H-shift barrier with respect to free RO + R'O vs reference barrier from literature SAR (Vereecken et al)**

**S3: Overall reaction energies (reaction products compared to the $^3$(RO···OR') reactant)**

**S4: Potential energy surfaces of the $^3$(RO···OR′) => ROH + R′$_{-H}$=O reactions for all systems studied here**

**S5: Calculated overall ISC rates for the studied systems**

**S6: Individual (state-specific) ISC rates for the studied systems**

## S1: H-shift barrier trends by grouping all donor and acceptor RO

Table S1.1: H-shift barriers grouped by the donor RO, with their standard deviations (SD).

| MeO as Donor | EtO as Donor | *i*PrO as Donor | AceO as Donor |
|---|---|---|---|
| 5.033 | 6.819 | 4.527 | 7.314 |
| 5.205 | 5.081 | 3.686 | 3.293 |
| 5.244 | 4.283 | 2.892 | 5.979 |
| 4.885 | 6.021 | 4.865 | 4.481 |
| 6.096 | 9.643 | 8.327 | 5.211 |
| 6.682 | 6.743 | 4.648 | 7.630 |
| SD = 0.707 | SD = 1.853 | SD = 1.867 | SD = 1.668 |

Table S1.2: H-shift barriers grouped by the acceptor RO, with their standard deviations (SD).

| MeO as acceptor | EtO as Acceptor | *i*PrO as Acceptor | AceO as Aceptor |
|---|---|---|---|
| 5.033 | 6.819 | 4.527 | 7.314 |
| 5.081 | 5.205 | 5.244 | 4.885 |
| 3.686 | 2.892 | 4.283 | 6.021 |
| 3.293 | 5.979 | 4.481 | 4.865 |
| 5.666 | 4.096 | 5.262 | 7.179 |
| 5.686 | 4.718 | 5.441 | 5.703 |
| SD = 1.016 | SD = 1.388 | SD = 0.497 | SD = 1.071 |

**S2: H-shift barrier with respect to free RO + R'O vs reference barrier from literature SAR (Vereecken et al).**

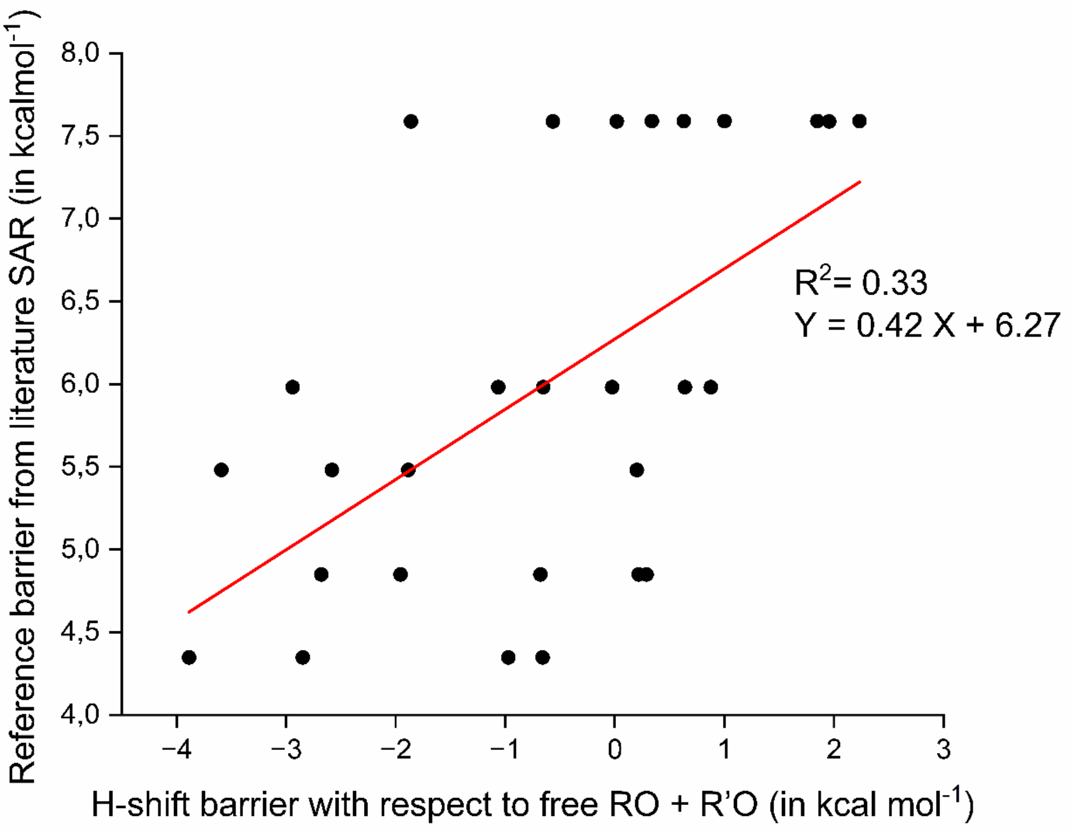


Figure S2.1: The computed H-shift barrier with respect to free RO + R'O vs the reference barrier computed using a literature SAR (Vereecken et al, see manuscript text for details).

Table S2: H-shift barriers with respect to the hypothetical isolated RO + R'O pair (in kcal/mol, with and without out zero-point energy (ZPE) corrections), at the DFT level. (Note that in real atmospheric conditions, collisions of free and isolated RO + R'O radicals will never happen – this data provided for reference and comparison only).

| $^3$(RO···OR′) cluster | | Barriers without ZPE corrections | Barriers including ZPE |
|---|---|---|---|
| MeO···OEt | $TS_1$ | 2.183 | 1.001 |
| | $TS_2$ | 1.753 | 0.877 |
| MeO···O*i*Pr | $TS_1$ | 3.322 | 1.848 |
| | $TS_2$ | 0.872 | 0.290 |
| MeO···OAce | $TS_1$ | 3.059 | 2.232 |
| | $TS_2$ | 0.898 | 0.640 |
| MeO···OBuOH | $TS_1$ | 1.537 | 0.631 |
| | $TS_2$ | 0.680 | 0.200 |
| MeO···O*i*PrOH | $TS_1$ | 1.322 | 0.337 |
| | $TS_2$ | - 1.165 | - 0.659 |
| EtO···O*i*Pr | $TS_1$ | 1.218 | - 0.565 |
| | $TS_2$ | - 0.613 | -1.956 |
| EtO···OAce | $TS_1$ | 0.953 | 0.018 |
| | $TS_2$ | 1.457 | - 0.024 |
| EtO···OBuOH | $TS_1$ | 3.467 | 1.957 |
| | $TS_2$ | - 2.899 | - 3.591 |
| EtO···O*i*PrOH | $TS_1$ | - 0.479 | - 1.859 |
| | $TS_2$ | - 3.011 | - 3.884 |
| *i*prO···OAce | $TS_1$ | - 0.141 | - 0.679 |
| | $TS_2$ | 0.215 | -1.063 |
| *i*prO···OPrOH | $TS_1$ | 0.993 | 0.218 |
| | $TS_2$ | - 2.442 | - 2.847 |
| iprO···OBuOH | $TS_1$ | - 1.650 | - 2.678 |
| | $TS_2$ | - 1.139 | -1.884 |
| AceO···O*i*PrOH | $TS_1$ | - 1.969 | - 2.940 |
| | $TS_2$ | - 0.345 | - 0.971 |
| AceO···OBuOH | $TS_1$ | 0.311 | - 0.654 |
| | $TS_2$ | - 2.600 | - 2.580 |

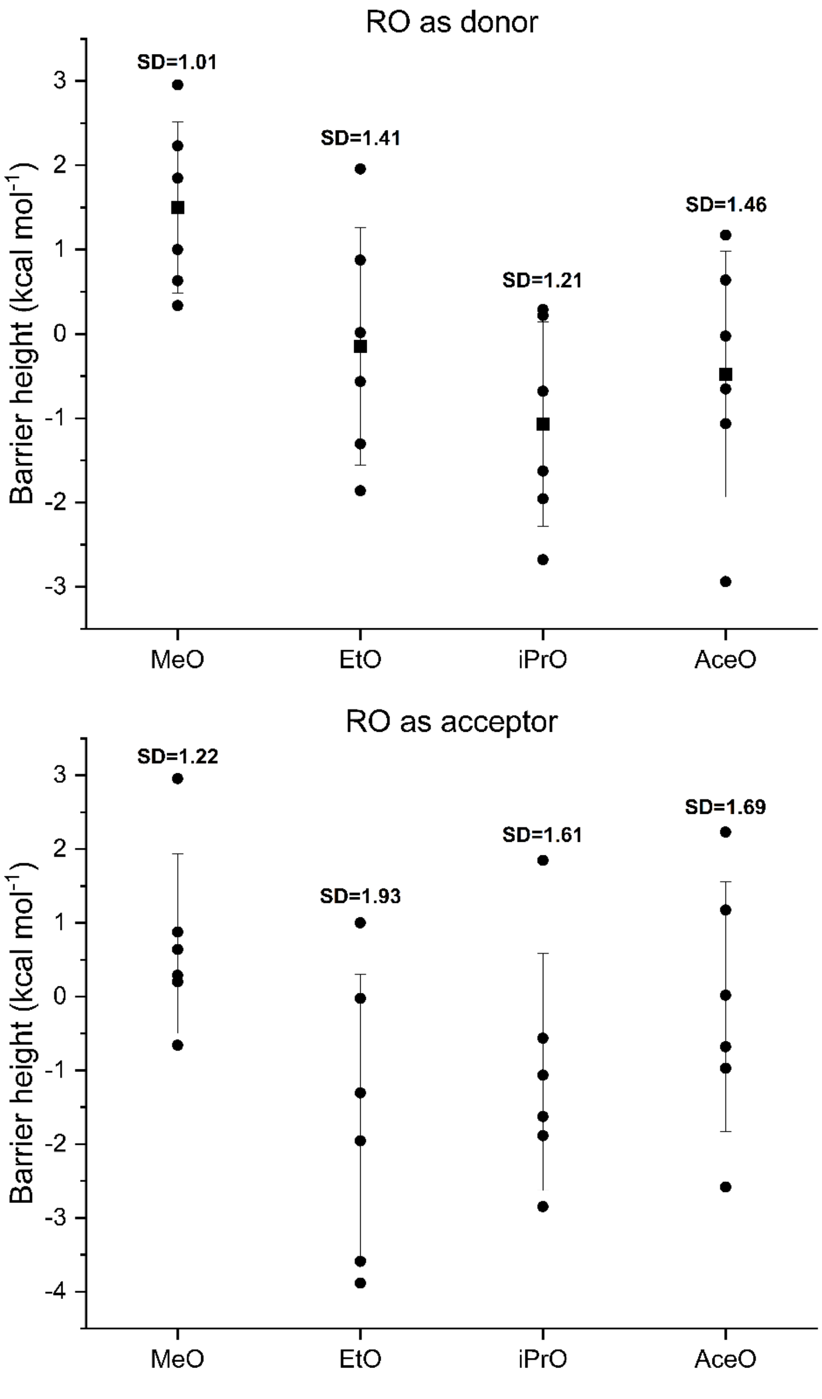


Figure S2.2: Top: H-shift barrier trends (with respect to free RO + R'O) by grouping all the donor RO. Bottom: H-shift barrier trends (with respect to free RO + R'O) by grouping all the acceptor RO. SD: standard deviation.

## S3: Overall reaction energies (reaction products compared to the $^3$(RO···OR') reactant)

Table S3.1: Reaction energies (electronic energies, not including zero-point corrections) for both sets of products combinations (triplet carbonyl and singlet alcohol and vice versa) for reaction channels $TS_1$ and $TS_2$, for all studied systems. As each system has two reaction channels, each with two possible product multiplicity combinations, there are four entries for each $^3$(RO···OR') reactant.

| $^3$(RO···OR') cluster | $TS_1$ Products | ΔE relative to $TS_1$ Product | $TS_2$ Products | ΔE relative to $TS_2$ Product |
|---|---|---|---|---|
| MeO···OEt | $^1$HCHO + $^3$EtOH | 84.892 | $^1CH_3CHO$ + $^3CH_3OH$ | # |
| | $^3$HCHO + $^1$EtOH | 1.197 | $^3CH_3CHO$ + $^1CH_3OH$ | -4.119 |
| MeO···O*i*Pr | $^1$HCHO+ $^3$*i*PrOH | 85.011 | $^1$Acetone + $^3CH_3$-OH | # |
| | $^3$HCHO+ $^1$*i*PrOH | 0.380 | $^3$Acetone + $^1CH_3$-OH | -8.810 |
| MeO···OAce | $^1$HCHO + $^3$Hydroxyacetone | 51.706 | $^1CH_3$-glyoxal + $^3CH_3$-OH | # |
| | $^3$HCHO + $^1$Hydroxyacetone | -2.042 | $^3CH_3$-glyoxal + $^1CH_3$-OH | -24.618 |
| MeO···OBuOH | $^1$HCHO + $^3$1,2-Butanediol | 57.543 | $^1$1-Hydroxy-2-butanone + $^3CH_3$-OH | # |
| | $^3$HCHO + $^1$1,2-Butanediol | 3.304 | $^3$1-Hydroxy-2-butanone + $^1CH_3$-OH | -5.097 |
| MeO···O*i*PrOH | $^1$HCHO + $^3$1,2-Propanediol | # | $^1$1-Hydroxy-acetone +$^3CH_3$-OH | # |
| | $^3$HCHO + $^1$1,2-Propanediol | 3.339 | $^3$1-Hydroxy-acetone +$^1CH_3$-OH | -5.230 |
| EtO···O*i*Pr | $^1CH_3CHO$ + $^3$Propanol | 27.352 | $^1$Acetone + $^3$EtOH | 22.501 |
| | $^3CH_3CHO$ + $^1$Propanol | -4.326 | $^3$Acetone + $^1$EtOH | -8.200 |
| EtO···OAce | $^1CH_3CHO$ + $^3$Hydroxyacetone | -4.822 | $^1CH_3$-glyoxal + $^3$EtOH | 33.326 |
| | $^3CH_3CHO$ + $^1$Hydroxyacetone | -5.618 | $^3CH_3$-glyoxal + $^1$EtOH | -22.879 |
| EtO···OBuOH | $^1CH_3CHO$ + $^3$1,2-Butanediol | -0.009 | $^1$1-Hydroxy-2-butanone + $^3$EtOH | 25.220 |
| | $^3CH_3CHO$ + $^1$1,2-Butanediol | -1.295 | $^3$1-Hydroxy-2-butanone + $^1$EtOH | -4.381 |
| EtO···O*i*PrOH | $^1CH_3CHO$ + $^3$1,2-Propaneediol | # | $^1$1-Hydroxy-acetone + $^3$EtOH | 25.423 |
| | $^3CH_3CHO$ + $^1$1,2-Propaneediol | -1.270 | $^3$1-Hydroxy-acetone + $^1$EtOH | -4.523 |
| *i*prO···OAce | $^1$Acetone + $^3$Hydroxyacetone | -8.836 | $^1CH_3$-glyoxal + $^3$Propanol | 34.164 |
| | $^3$Acetone + $^1$Hydroxyacetone | -9.591 | $^3CH_3$-glyoxal + $^1$Propanol | -22.977 |
| *i*prO···OPrOH | $^1$Acetone + $^3$1,2-Propanediol | # | $^1$1-Hydroxy-acetone + $^3$Propanol | 25.902 |
| | $^3$Acetone + $^1$1,2-Propanediol | -5.602 | $^3$1-Hydroxy-acetone + $^1$Propanol | -4.981 |
| *i*prO···OBuOH | $^1$Acetone + $^3$1,2-Butanediol | -4.165 | $^1$1-Hydroxy-2-butanone + $^3$Propanol | 25.915 |
| | $^3$Acetone + $^1$1,2-Butanediol | -5.412 | $^3$1-Hydroxy-2-butanone + $^1$Propanol | -4.622 |
| AceO···O*i*PrOH | $^1$Methyl-glyoxal + $^3$1,2-propanediol | # | $^1$1-Hydroxy-acetone + $^3$1-Hydroxy-acetone | -6.424 |
| | $^3$Methyl-glyoxal + $^1$1,2-propanediol | -20.431 | $^3$1-Hydroxy-acetone + $^1$1-Hydroxy-acetone | -6.424 |
| AceO···OBuOH | $^1$Methyl-glyoxal + $^3$1,2-butanediol | 7.469 | $^1$1-Hydroxy-2-butanone + $^3$1-Hydroxy-acetone | -5.450 |
| | $^3$Methyl-glyoxal + $^1$1,2-butanediol | -19.280 | $^3$1-Hydroxy-2-butanone + $^1$1-Hydroxy-acetone | -5.105 |

$^{\#}$ 1,2-propanediol and methanol ($CH_3$-OH) could not be optimized in the triplet state.

**S4: Potential energy surfaces of the $^3$(RO···OR′) => ROH + R′$_{-H}$=O reaction for all systems studied here.**

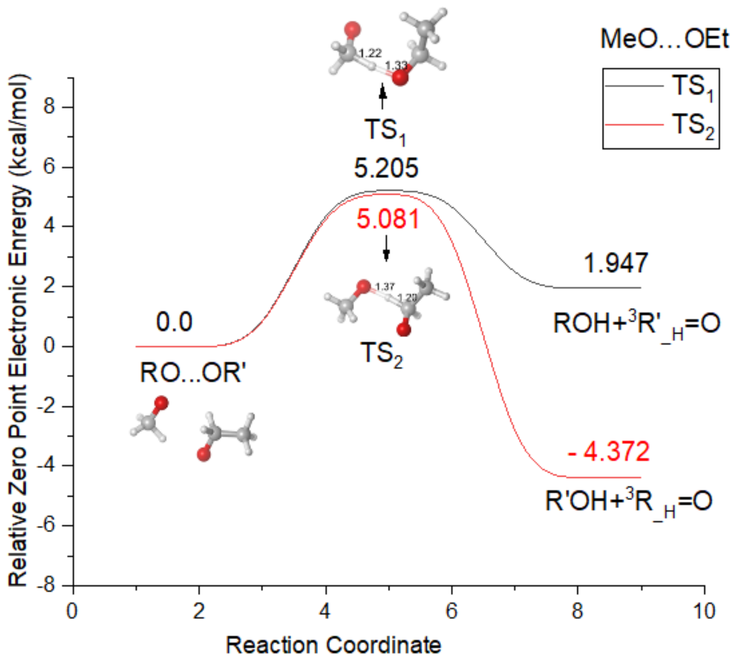


Figure S4.1: Potential energy surface of the $^3$(RO···OR′): ROH + R′$_{-H}$=O reaction for the MeO···OEt system studied here, calculated at the *ω*B97X-D/aug-cc-pVTZ level, including zero-point vibrational energy.

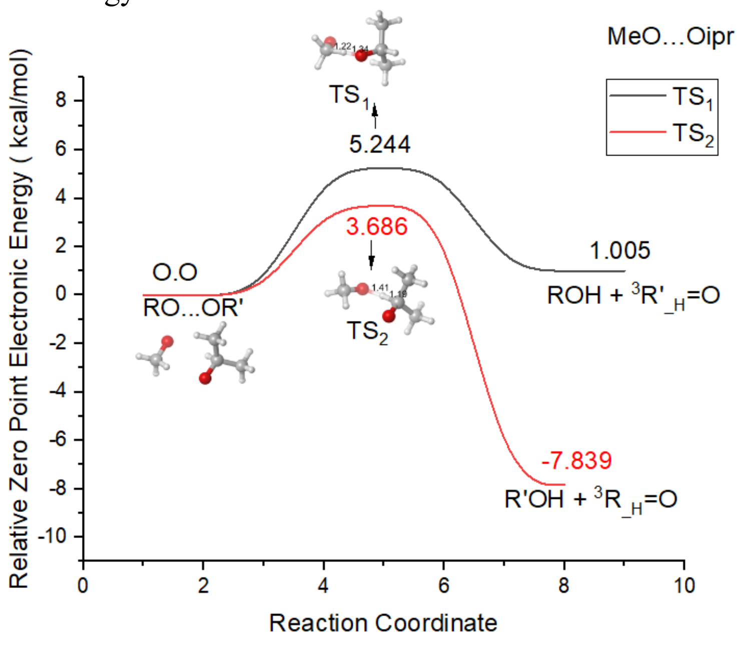


Figure S4.2: Potential energy surface of the $^3$(RO···OR′): ROH + R′$_{-H}$=O reaction for the MeO···O*i*Pr system studied here, calculated at the *ω*B97X-D/aug-cc-pVTZ level, including zero-point vibrational energy.

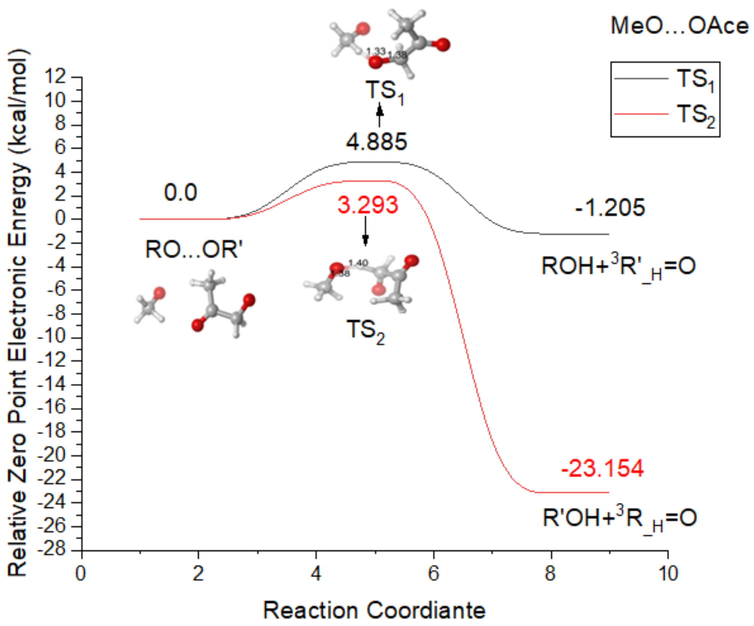


Figure S4.3: Potential energy surface of the $^3$(RO···OR′): ROH + R′−H=O reaction for the MeO···OAce system studied here, calculated at the ωB97X-D/aug-cc-pVTZ level, including zero-point vibrational energy.

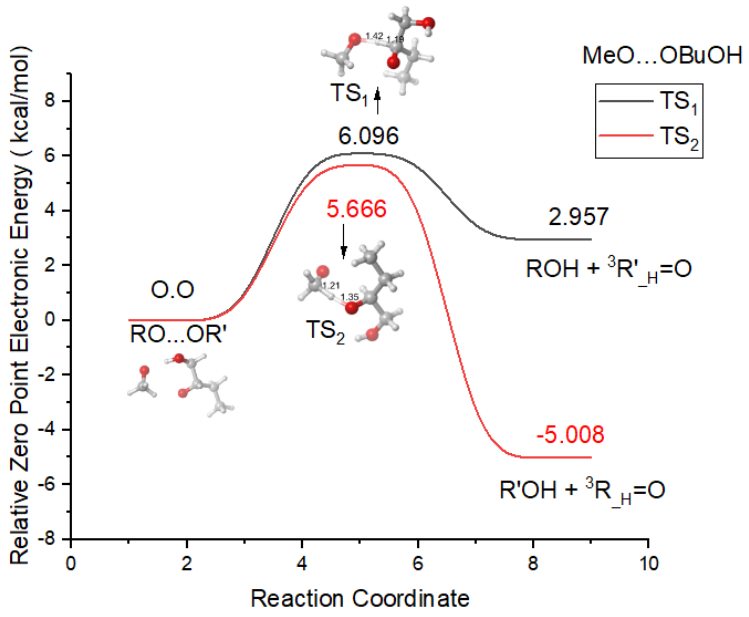


Figure S4.4: Potential energy surface of the $^3$(RO···OR′): ROH + R′−H=O reaction for the MeO···OBuOH system studied here, calculated at the ωB97X-D/aug-cc-pVTZ level, including zero-point vibrational energy.

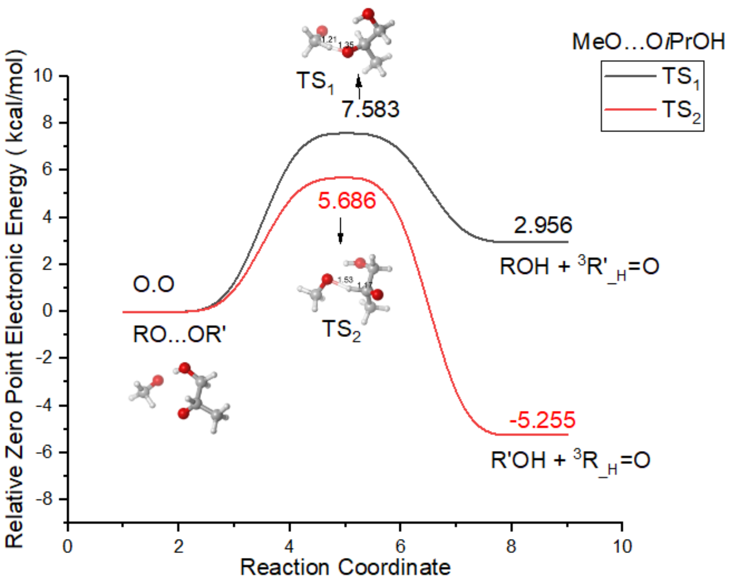


Figure S4.5: Potential energy surface of the $^3$(RO$\cdots$OR′): ROH + R′$_{-H}$=O reaction for the MeO$\cdots$O*i*PrOH system studied here, calculated at the *ω*B97X-D/aug-cc-pVTZ level, including zero-point vibrational energy.

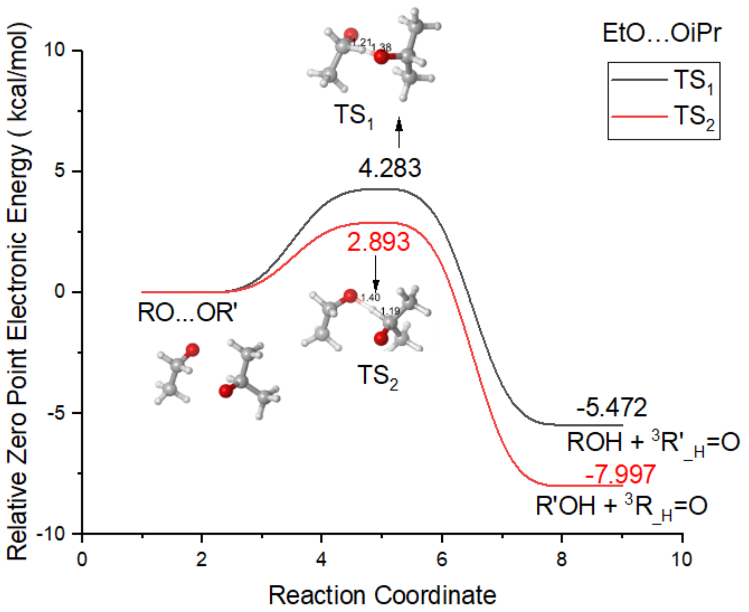


Figure S4.6: Potential energy surface of the $^3$(RO$\cdots$OR′): ROH + R′$_{-H}$=O reaction for the EtO$\cdots$O*i*Pr system studied here, calculated at the *ω*B97X-D/aug-cc-pVTZ level, including zero-point vibrational energy.

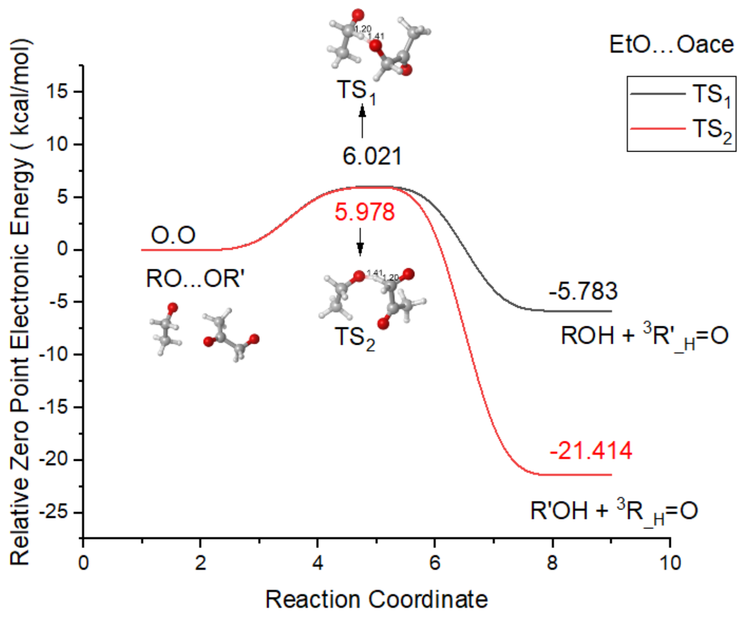


Figure S4.7: Potential energy surface of the $^3$(RO···OR′): ROH + $R'_{-H}$=O reaction for the MeO···OAce system studied here, calculated at the $\omega$B97X-D/aug-cc-pVTZ level, including zero-point vibrational energy.

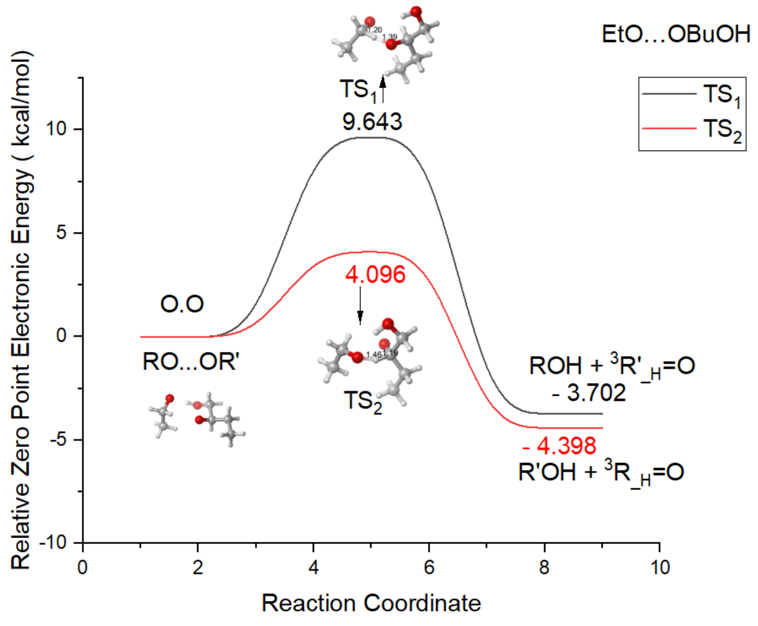


Figure S4.8: Potential energy surface of the $^3$(RO···OR′): ROH + $R'_{-H}$=O reaction for the EtO···OBuOH system studied here, calculated at the $\omega$B97X-D/aug-cc-pVTZ level, including zero-point vibrational energy.

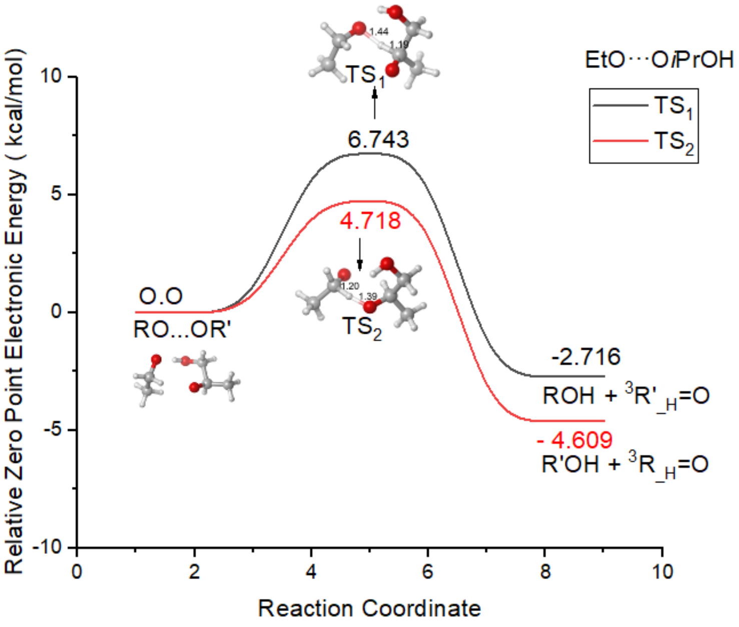


Figure S4.9: Potential energy surface of the $^3$(RO···OR′): ROH + R′$_{-H}$=O reaction for the EtO···O*i*PrOH system studied here, calculated at the *ω*B97X-D/aug-cc-pVTZ level, including zero-point vibrational energy.

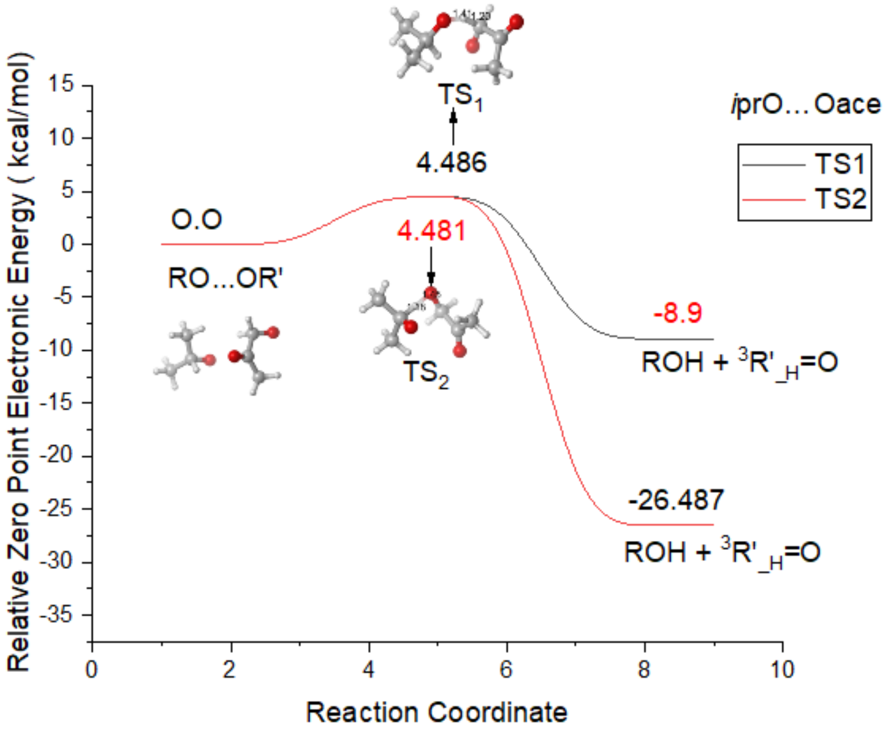


Figure S4.10: Potential energy surface of the $^3$(RO···OR′): ROH + R′$_{-H}$=O reaction for the *i*PrO···OAce system studied here, calculated at the *ω*B97X-D/aug-cc-pVTZ level, including zero-point vibrational energy.

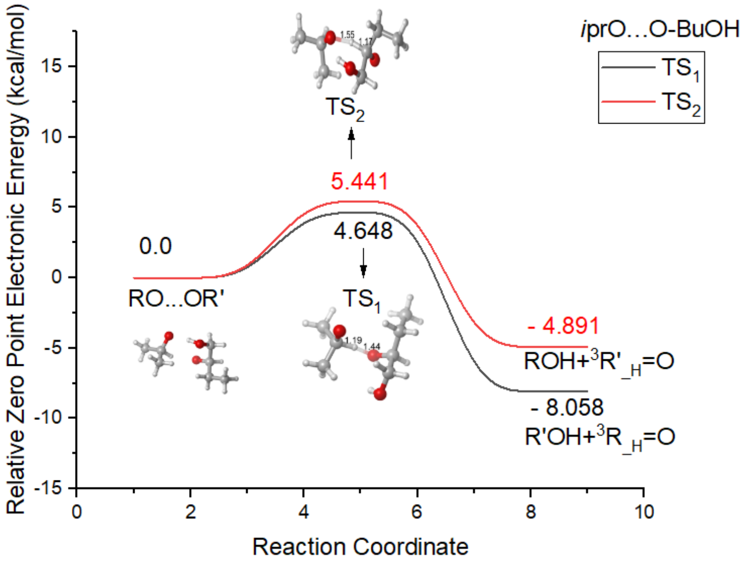


Figure S4.11: Potential energy surface of the $^3$(RO···OR′): ROH + $R'_{-H}$=O reaction for the *i*PrO···OBuOH system studied here, calculated at the *ω*B97X-D/aug-cc-pVTZ level, including zero-point vibrational energy.

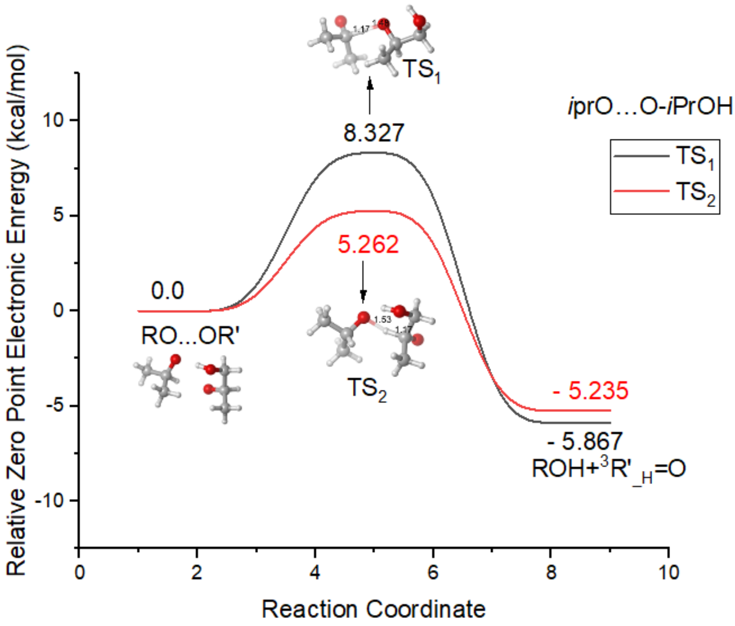


Figure S4.12: Potential energy surface of the $^3$(RO···OR′): ROH + $R'_{-H}$=O reaction for the *i*PrO···O-*i*PrOH system studied here, calculated at the *ω*B97X-D/aug-cc-pVTZ level, including zero-point vibrational energy.

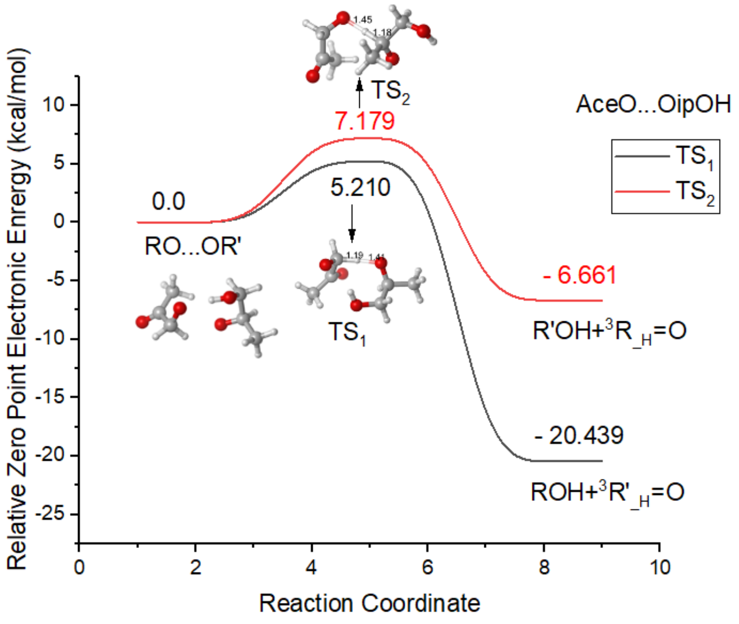


Figure S4.13: Potential energy surface of the ³(RO···OR′): ROH + R′–H=O reaction for the AceoO···O*i*PrOH system studied here, calculated at the *ω*B97X-D/aug-cc-pVTZ level, including zero-point vibrational energy.

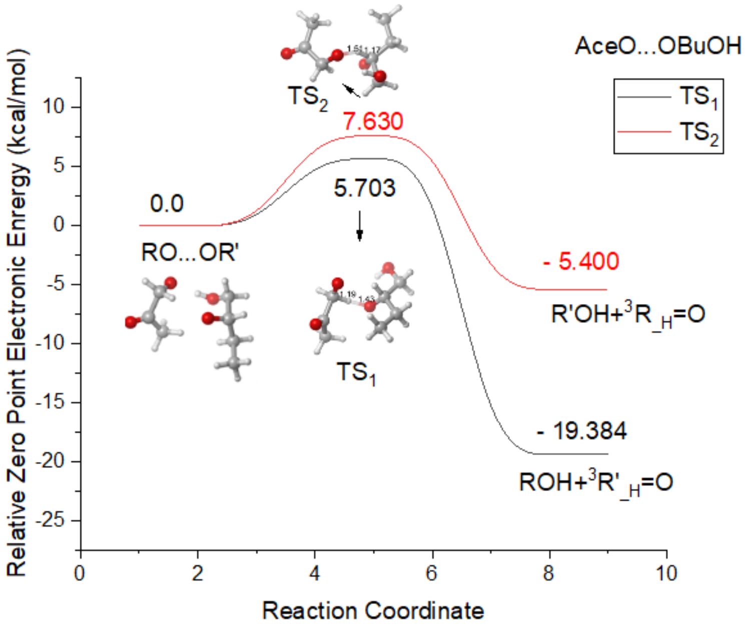


Figure S4.14: Potential energy surface of the ³(RO···OR′): ROH + R′–H=O reaction for the AceoO···OBuOH system studied here, calculated at the *ω*B97X-D/aug-cc-pVTZ level, including zero-point vibrational energy.

## S5: Calculated overall ISC rates for the studied systems.

| $^3$(RO···OR') cluster | Σ $k_{rISC}$ ($s^{-1}$) | O···O distance in (Å) |
|---|---|---|
| MeO···OEt | $2.28 \times 10^9$ | 3.493 |
| MeO···O*i*Pr | $2.05 \times 10^9$ | 3.470 |
| MeO···Oace | $2.28 \times 10^9$ | 6.140 |
| MeO···OBuOH | $7.90 \times 10^9$ | 3.347 |
| MeO···O*i*PrOH | $7.93 \times 10^9$ | 3.350 |
| EtO···O*i*Pr | $4.14 \times 10^9$ | 3.587 |
| EtO···OAce | $1.50 \times 10^9$ | 4.207 |
| EtO···OBuOH | $1.35 \times 10^9$ | 3.271 |
| EtO···O*i*PrOH | $1.29 \times 10^9$ | 3.272 |
| *i*PrO···OAce | $9.02 \times 10^8$ | 4.163 |
| *i*PrO···O*i*PrOH | $2.95 \times 10^9$ | 3.314 |
| *i*PrO···OBuOH | $6.33 \times 10^9$ | 3.325 |
| AceO···O*i*PrOH | $1.01 \times 10^8$ | 3.424 |
| AceO···OBuOH | $1.11 \times 10^9$ | 3.426 |

## S6: Individual (state-specific) ISC rates for the studied systems

**Table S6(a)**: The SOCME ($cm^{-1}$), Energy Gap ($cm^{-1}$), and $k_{ISC}$ ($s^{-1}$) computed for the $^3$(MeO···OEt) cluster.

| Transition | SOC | Energy Gap | Σ $k_{rISC}$ ($s^{-1}$) |
|---|---|---|---|
| $T_1 \rightarrow S_1$ | 0 | 261.42 | $0.00 \times 10^0$ |
| $T_1 \rightarrow S_2$ | 114 | 1555.332 | $2.22 \times 10^9$ |
| $T_1 \rightarrow S_3$ | 87.73 | 2112.108 | $5.56 \times 10^7$ |
| $T_1 \rightarrow S_4$ | 0 | 3538.146 | $0.00 \times 10^0$ |
| | | Total rate | $2.28 \times 10^9$ |

**Table S6(b)**: The SOCME ($cm^{-1}$), Energy Gap ($cm^{-1}$), and $k_{ISC}$ ($s^{-1}$) computed for the $^3$(MeO···O*i*Pr) cluster.

| Transition | SOC | Energy Gap | Σ $k_{rISC}$ ($s^{-1}$) |
|---|---|---|---|
| $T_1 \rightarrow S_1$ | 0.38 | 0 | $2.31 \times 10^8$ |
| $T_1 \rightarrow S_2$ | 117.94 | 1641.061 | $1.46 \times 10^9$ |
| $T_1 \rightarrow S_3$ | 84.57 | 1772.062 | $3.57 \times 10^8$ |
| $T_1 \rightarrow S_4$ | 0.31 | 3293.155 | $4.21 \times 10^{-1}$ |
| | | Total rate | $2.05 \times 10^9$ |

**Table S6(c)**: The SOCME ($cm^{-1}$), Energy Gap ($cm^{-1}$), and $k_{ISC}$ ($s^{-1}$) computed for the $^3$(MeO···OAce) cluster.

| Transition | SOC | Energy Gap | Σ $k_{rISC}$ ($s^{-1}$) |
|---|---|---|---|
| $T_1 \rightarrow S_1$ | 0 | 261.401 | $0.00 \times 10^0$ |
| $T_1 \rightarrow S_2$ | 114 | 1555.314 | $2.22 \times 10^9$ |
| $T_1 \rightarrow S_3$ | 87.73 | 2112.088 | $5.56 \times 10^7$ |
| $T_1 \rightarrow S_4$ | 0 | 3538.132 | $0.00 \times 10^0$ |
| | | Total rate | $2.28 \times 10^9$ |

**Table S6(d)**: The SOCME ($cm^{-1}$), Energy Gap ($cm^{-1}$), and $k_{ISC}$ ($s^{-1}$) computed for the $^3$(MeO···OBuOH) cluster.

| Transition | SOC | Energy Gap | Σ $k_{rISC}$ ($s^{-1}$) |
|---|---|---|---|
| $T_1 \rightarrow S_1$ | 2.68 | 78.17 | $7.88 \times 10^9$ |
| $T_1 \rightarrow S_2$ | 102.43 | 2418.067 | $1.33 \times 10^7$ |
| $T_1 \rightarrow S_3$ | 106.73 | 2738.607 | $2.33 \times 10^6$ |
| $T_1 \rightarrow S_4$ | 2.28 | 5100.303 | $2.62 \times 10^{-4}$ |
| | | Total rate | $7.90 \times 10^9$ |

**Table S6(e)**: The SOCME ($cm^{-1}$), Energy Gap ($cm^{-1}$), and $k_{ISC}$ ($s^{-1}$) computed for the $^3$(MeO···O*i*PrOH) cluster.

| Transition | SOC | Energy Gap | Σ $k_{rISC}$ ($s^{-1}$) |
|---|---|---|---|
| $T_1 \rightarrow S_1$ | 2.66 | 74.323 | $7.91 \times 10^9$ |
| $T_1 \rightarrow S_2$ | 102.66 | 2366.445 | $1.79 \times 10^7$ |
| $T_1 \rightarrow S_3$ | 106.67 | 2732.238 | $2.42 \times 10^6$ |
| $T_1 \rightarrow S_4$ | 2.21 | 5047.79 | $3.31 \times 10^{-4}$ |
| | | Total rate | $7.93 \times 10^9$ |

**Table S6(f)**: The SOCME ($cm^{-1}$), Energy Gap ($cm^{-1}$), and $k_{ISC}$ ($s^{-1}$) computed for the $^3$(EtO···O*i*Pr) cluster.

| Transition | SOC | Energy Gap | Σ $k_{rISC}$ ($s^{-1}$) |
|---|---|---|---|
| $T_1 \rightarrow S_1$ | 2.56 | 299.846 | $2.47 \times 10^9$ |
| $T_1 \rightarrow S_2$ | 109.1 | 1591.825 | $1.66 \times 10^9$ |
| $T_1 \rightarrow S_3$ | 96.64 | 2373.81 | $1.52 \times 10^7$ |
| $T_1 \rightarrow S_4$ | 1.84 | 3760.95 | $1.04 \times 10^0$ |
| | | Total rate | $4.14 \times 10^9$ |

**Table S6(g)**: The SOCME ($cm^{-1}$), Energy Gap ($cm^{-1}$), and $k_{ISC}$ ($s^{-1}$) computed for the $^3$(EtO···OAce) cluster.

| Transition | SOC | Energy Gap | Σ $k_{rISC}$ ($s^{-1}$) |
|---|---|---|---|
| $T_1 \rightarrow S_1$ | 0.02 | 300.227 | $1.50 \times 10^5$ |
| $T_1 \rightarrow S_2$ | 103.54 | 1592.374 | $1.49 \times 10^9$ |
| $T_1 \rightarrow S_3$ | 106.65 | 2373.662 | $1.86 \times 10^7$ |
| $T_1 \rightarrow S_4$ | 0.39 | 3761.362 | $4.65 \times 10^{-2}$ |
| | | Total rate | $1.50 \times 10^9$ |

**Table S6(h)**: The SOCME ($cm^{-1}$), Energy Gap ($cm^{-1}$), and $k_{ISC}$ ($s^{-1}$) computed for the $^3$(EtO···OBuOH) cluster.

| Transition | SOC | Energy Gap | Σ $k_{rISC}$ ($s^{-1}$) |
|---|---|---|---|
| $T_1 \rightarrow S_1$ | 0.92 | 2.789 | $1.34 \times 10^9$ |
| $T_1 \rightarrow S_2$ | 102.34 | 2394.722 | $1.52 \times 10^7$ |
| $T_1 \rightarrow S_3$ | 107.37 | 2773.062 | $1.94 \times 10^6$ |
| $T_1 \rightarrow S_4$ | 1.32 | 5232.735 | $4.13 \times 10^{-5}$ |
| | | Total rate | $1.35 \times 10^9$ |

**Table S5(i)**: The SOCME ($cm^{-1}$), Energy Gap ($cm^{-1}$), and $k_{ISC}$ ($s^{-1}$) computed for the $^3$(EtO···O*i*PrOH) cluster.

| Transition | SOC | Energy Gap | Σ $k_{rISC}$ ($s^{-1}$) |
|---|---|---|---|
| $T_1 \rightarrow S_1$ | 0.9 | 4.042 | $1.27 \times 10^9$ |
| $T_1 \rightarrow S_2$ | 102.56 | 2367.944 | $1.77 \times 10^7$ |
| $T_1 \rightarrow S_3$ | 107.27 | 2775.727 | $1.91 \times 10^6$ |
| $T_1 \rightarrow S_4$ | 1.44 | 5205.169 | $5.75 \times 10^{-5}$ |
| | | Total rate | $1.29 \times 10^9$ |

**Table S6(j)**: The SOCME ($cm^{-1}$), Energy Gap ($cm^{-1}$), and $k_{ISC}$ ($s^{-1}$) computed for the $^3$(*i*PrO···Oace) cluster.

| Transition | SOC | Energy Gap | Σ $k_{rISC}$ ($s^{-1}$) |
|---|---|---|---|
| $T_1 \rightarrow S_1$ | 0.64 | 121.737 | $3.64 \times 10^8$ |
| $T_1 \rightarrow S_2$ | 104.66 | 1776.17 | $5.34 \times 10^8$ |
| $T_1 \rightarrow S_3$ | 105.05 | 2671.533 | $3.31 \times 10^6$ |
| $T_1 \rightarrow S_4$ | 0.87 | 4303.572 | $3.53 \times 10^{-3}$ |
| | | Total rate | $9.02 \times 10^8$ |

**Table S6(k)**: The SOCME ($cm^{-1}$), Energy Gap ($cm^{-1}$), and $k_{ISC}$ ($s^{-1}$) computed for the $^3$(*i*PrO···O*i*PrOH) cluster.

| Transition | SOC | Energy Gap | Σ $k_{rISC}$ ($s^{-1}$) |
|---|---|---|---|
| $T_1 \rightarrow S_1$ | 1.64 | 80.482 | $2.92 \times 10^9$ |
| $T_1 \rightarrow S_2$ | 102.9 | 2269.506 | $3.12 \times 10^7$ |
| $T_1 \rightarrow S_3$ | 106.1 | 2904.598 | $4.49 \times 10^5$ |
| $T_1 \rightarrow S_4$ | 2.2 | 5141.762 | $1.92 \times 10^{-4}$ |
| | | Total rate | $2.95 \times 10^9$ |

**Table S6(l)**: The SOCME ($cm^{-1}$), Energy Gap ($cm^{-1}$), and $k_{ISC}$ ($s^{-1}$) computed for the $^3$(iprO···OBuOH) cluster.

| Transition | SOC | Energy Gap | Σ $k_{rISC}$ ($s^{-1}$) |
|---|---|---|---|
| $T_1 \rightarrow S_1$ | 3.18 | 195.398 | $6.30 \times 10^9$ |
| $T_1 \rightarrow S_2$ | 102.93 | 2269.984 | $3.12 \times 10^7$ |
| $T_1 \rightarrow S_3$ | 105.74 | 3121.925 | $1.30 \times 10^5$ |
| $T_1 \rightarrow S_4$ | 1.02 | 5243.17 | $2.32 \times 10^5$ |
| | | Total rate | $6.33 \times 10^9$ |

**Table S6(m)**: The SOCME ($cm^{-1}$), Energy Gap ($cm^{-1}$), and kISC ($s^{-1}$) computed for the $^3$(AceO···O*i*PrOH) cluster.

| Transition | SOC | Energy Gap | Σ $k_{rISC}$ ($s^{-1}$) |
|---|---|---|---|
| $T_1 \rightarrow S_1$ | 0.31 | 92.815 | $9.83 \times 10^7$ |
| $T_1 \rightarrow S_2$ | 108.44 | 2731.937 | $2.50 \times 10^6$ |
| $T_1 \rightarrow S_3$ | 97.27 | 3552.136 | $9.50 \times 10^3$ |
| $T_1 \rightarrow S_4$ | 2.56 | 6147.978 | $2.13 \times 10^{-7}$ |
| | | Total rate | $1.01 \times 10^8$ |

**Table S6(n):** The SOCME ($cm^{-1}$), Energy Gap ($cm^{-1}$), and $k_{ISC}$ ($s^{-1}$) computed for the $^3$(AceO···OBuOH) cluster.

| Transition | SOC | Energy Gap | $\Sigma\ k_{rISC}$ ($s^{-1}$) |
|---|---|---|---|
| $T_1 \rightarrow S_1$ | 0.83 | 5 | $1.10 \times 10^9$ |
| $T_1 \rightarrow S_2$ | 103.84 | 2642.074 | $3.83 \times 10^6$ |
| $T_1 \rightarrow S_3$ | 102.04 | 3757.974 | $3.24 \times 10^3$ |
| $T_1 \rightarrow S_4$ | 2.34 | 6305.763 | $7.27 \times 10^{-8}$ |
| | | Total rate | $1.11 \times 10^9$ |
